# Does immediate dentin sealing influence postoperative sensitivity in teeth restored with indirect restorations? A systematic review and meta‐analysis

**DOI:** 10.1111/jerd.12841

**Published:** 2021-12-03

**Authors:** Uros Josic, Maicon Sebold, Rodrigo B. E. Lins, Jelena Savovic, Claudia Mazzitelli, Tatjana Maravic, Annalisa Mazzoni, Lorenzo Breschi

**Affiliations:** ^1^ Department of Biomedical and Neuromotor Sciences University of Bologna Bologna; ^2^ Clinic for Pediatric and Preventive Dentistry, School of Dental Dedicine University of Belgrade Belgrade Serbia; ^3^ Department of Restorative Dentistry, Operative Dentistry Devision, Piracicaba Dental School University of Campinas Piracicaba Brazil; ^4^ Dentistry Course State University of Paraiba Araruna Brazil; ^5^ Population Health Sciences, Bristol Medical School University of Bristol Bristol UK

**Keywords:** immediate dentin sealing, indirect restoration, systematic review

## Abstract

**Objective:**

This study comprehensively reviewed clinical trials that investigated the effect of immediate dentin sealing (IDS) technique on postoperative sensitivity (POS) and clinical performance of indirect restorations.

**Materials and methods:**

The systematic review was conducted according to the preferred reporting items for systematic reviews and meta‐analyses statement, and was guided by the PICOS strategy. Clinical trials in which adult patients received at least one indirect restoration cemented with IDS approach and one restoration cemented following the delayed dentin sealing (DDS) were considered.

**Results:**

Following title screening and full‐text reading, four studies met the inclusion criteria and were included for qualitative synthesis, while two studies were selected for quantitative synthesis. According to Risk of bias‐2 tool, two studies were classified as “some concerns” for the outcome POS. No statistically significant differences were found between teeth restored with indirect restorations using the IDS and DDS approach for POS (*p* > 0.05), neither at the baseline (very low certainty of evidence according to GRADE) nor after 2 years of follow‐up (low certainty of evidence according to GRADE).

**Conclusion:**

There is low‐certainty evidence that IDS does not reduce POS in teeth restored with indirect restorations.

**Clinical significance:**

There is no clinical evidence to favor IDS over DDS when restoring teeth with indirect restorations.

## 1 INTRODUCTION

The use of advanced adhesive systems and resin cements has allowed dentists to restore posterior teeth with large defects using a minimally invasive approach, without the need to rely on retentive tooth preparation form and/or perform root canal treatment.^1^ However, delaying endodontic treatment in cases where a thin layer of dentin is present can result in postoperative sensitivity (POS), causing patient's dissatisfaction due to difficulties in resolving pain‐related conditions.^2^, ^3^ A recent systematic review found that the 5‐year survival rate for complete and partial crowns is more than 90%, and that one of the most common biological complications is tooth hypersensitivity and/or pulpitis, which leads to endodontic treatment, regardless of the type and material of the restoration.^4^ Similarly, another systematic review reported an acceptable 3‐year survival rate for inlay‐retained fixed dental prostheses, with dentin sensitivity being once more identified as one of the primary complications.^5^

When a tooth needs to be prepared to receive a fixed prosthesis, a series of consecutive clinical steps should be performed, starting from biomechanical preparation to the cementation procedure, and each of these steps can be a potential cause of POS.^6^ The relationship between the clinical steps and the incidence of POS has been widely investigated, with special emphasis on luting cements since these have been considered to have an important role in the pathogenesis of POS.^2^

Considering various cementation strategies, no differences in terms of retention have been observed between conventional luting procedures with glass‐ionomer, resin‐modified glass‐ionomer, or zinc phosphate cements compared to adhesive cementation with resin cements when used for luting zirconia or lithium disilicate crowns.^7^ Self‐adhesive resin cements have become increasingly popular among dentists due to their user friendliness, decreased mismanagement possibilities and, as claimed by manufacturers, reduced POS. Interestingly, when self‐adhesive resin cements have been used for luting glass–ceramic restorations in posterior teeth, no clinical differences were found after 1‐year of clinical service compared to conventional (multistep) resin cements.^8^ However, this observation period may be regarded as short‐term, and after a follow‐up of 5 and 10 years, the most common types of failure reported were fractures/chipping (4%) of ceramic and composite indirect restorations, followed by endodontic complications (3%), secondary caries (1%), and debonding (1%).^9^

In an attempt to overcome functional and biological complications, applying a thin layer of a coating material or dentin bonding system with flowable composite resin on both enamel and dentin immediately after tooth preparation was suggested by Japanese clinicians in the early 1990s. This technique is known as resin‐coating technique.^10^ Subsequently, Magne et al. reported a similar approach, in which the application of dentin bonding agent is limited only to the exposed dentin and this approach became known as immediate dentin sealing (IDS).^11^ Resin‐coating technique and IDS are especially indicated to protect the pulp after preparation for indirect restoration, since pulp is indirectly exposed due to the presence of dentinal tubules, which connects it to the exposed surface. Compared to conventional temporary sealing materials, dentin bonding agents have better sealing properties and may protect dentin and pulp physically, chemically, and biologically.^12^ The use of both techniques also prevents the collapse of hybrid layers prior to their polymerization, eliminates POS, favors the development of stress‐free dentin bonds, and it may result in improved margin adaptation.^11^, ^13^ Overall, when comparing IDS and delayed dentin sealing (DDS, absence of IDS), literature reports higher bond strengths of indirect restorations when IDS is performed, with both etch‐and‐rinse and self‐etch adhesive systems showing a beneficial effect on the bond strength.^14^ Moreover, it has been shown that besides etch‐and‐rinse and self‐etch adhesives, IDS can be successfully performed using universal adhesives in combination with CAD/CAM ceramics used as indirect restoration.^15^, ^16^ Although approximately 30 years have passed since the first reported study on IDS, the majority of conclusions related to the IDS technique was drawn from in vitro studies, while clinical trials on this subject have only recently become available in literature. Therefore, the aim of this article was to answer the following question: does IDS have an influence on POS and clinical parameters of indirect restorations? The primary outcome analyzed was POS, while the secondary outcome of interest were survival rates and clinical parameters used for assessing indirect restorations (retention, marginal adaptation/discoloration, surface texture, color, recurrent caries, anatomic shape, and pulp vitality).

## 2 MATERIALS AND METHODS

### 2.1 Study protocol and registration

This study protocol was registered in the International Prospective Register of Systematic Reviews (PROSPERO) database under the number CRD 42020184902. The reporting of this systematic review and meta‐analysis followed the preferred reporting items for systematic reviews and meta‐analyses (PRISMA).^17^

### 2.2 Eligibility criteria and search strategy

The PICOS strategy^18^ that guided the choice of the inclusion criteria and informed the search strategy is described herein:

Population (P)–adult patients with the need of indirect restorations (inlays, onlays, overlays, crowns, or fixed partial dentures);

Intervention (I)–indirect restorations placed following the IDS technique;

Comparison (C)–indirect restorations placed following the DDS technique (without IDS);

Outcome (O)–clinical parameters used to evaluate indirect restorations (retention, marginal adaptation/discoloration, surface texture, color, POS, recurrent caries, anatomic shape, pulp vitality), and overall survival rates for different follow‐up periods. The “outcome” criteria were not applied to the search strategy, as they would limit the number of retrieved studies;

Study design (S)–clinical studies (prospective, retrospective, and randomized controlled clinical trials). The “study design” criterion was used for the search strategy to avoid the inclusion of a high number of laboratory studies.

The electronic search was carried out during the first week of June 2020 in the following databases: PubMed, Web of Science, Scopus, Clinical Trials, Virtual Health Library (VHL) LILACS, Cochrane Library, ReBEC, Open Gray, and Embase. The MeSH terms, synonyms, and free keywords that were used are summarized in Table S1. Additional manual search was carried out through the reference lists of the included articles to identify studies that had not been retrieved in the electronic search of the databases. No restriction of language or date, as well as no filters, was applied.

The exclusion criteria were as follows: (1) In vitro or ex vivo studies; (2) reviews (narrative or systematic); (3) case reports; (4) conference abstracts; (5) studies that did not present at least two groups of indirect restorations comparing IDS with DDS; (6) studies dealing only with direct restorations or comparing indirect restorations with direct restorations; (7) studies that compared outcomes between vital and non‐vital teeth; (8) studies on primary dentition; and (9) experiments carried out with animal subjects. No follow‐up period threshold was established for this systematic review and meta‐analysis, since POS, which is very likely to occur in the first hours or days after the restorative procedure,^19^ was the main outcome of interest.

### 2.3 Study selection and data extraction

References were retrieved from the databases mentioned above using the EndNote X9 software (Clarivate Analytics, Philadelphia, PA, USA). After the removal of duplicates by the software, the titles and abstracts of all retrieved papers were screened by two independent investigators (Maicon Sebold and Uros Josic). Articles that could potentially be included in the review were read in full to determine their eligibility. Disagreements between the two reviewers were solved by consulting with a third investigator (Annalisa Mazzoni)

Data extraction was performed by two independent investigators (Maicon Sebold and Uros Josic) using customized extraction forms in MS Word. We extracted details of the study (author, year, location, and study design), participants (number and age range), indirect restorations (number, type, and material used for indirect restorations, and type of teeth restored), dentin sealing approach (type of adhesive system used during restorative procedures, number of restorations placed with immediate or delayed dentin sealing), methodology (evaluation criteria, follow‐up periods, and overall survival rates), and results (success and failure rates, as well as statistical analyses). If essential data was not reported in a certain study, the corresponding author of that paper was contacted by email in an attempt to retrieve the necessary information. If no response was obtained after trying to contact the authors three times by email, the study was excluded from the review.

### 2.4 Risk of bias assessment

Quality and risk of bias of the eligible studies were assessed by three independent investigators (Maicon Sebold, Uros Josic, and Jelena Savovic). The revised Cochrane Collaboration's tool for assessing risk of bias in randomized clinical trials (RoB 2) was used.^20^ For nonrandomized clinical trials, risk of bias was assessed by the ROBINS‐I tool.^21^ The evaluators compared and discussed the data extracted from the selected studies, and a third investigator (Jelena Savovic) was consulted when necessary.

The RoB 2 tool^20^ contains algorithms that map responses to signaling questions regarding a proposed risk of bias judgment for each outcome assessed in a given study. Therefore, assessment criteria were divided into five domains: (1) risk of bias from randomization process; (2) bias due to deviations from intended interventions; (3) bias due to missing outcome data; (4) bias in measurement of the outcome; and (5) risk of bias in selection of the reported result. The risk of bias judgment for each of the five domains was classified as “low risk of bias,” “some concerns,” or “high risk of bias.” The overall risk of bias for a specific outcome of a certain study was determined according to the classification of the assessment criteria domains, following recommendations described in the RoB 2 tool. If at least one domain was rated as “some concerns” and all other domains “low risk,” the overall risk of bias could be “some concerns.” If several domains were rated as “some concerns,” the overall risk of bias could be either “some concerns” or “high,” depending on the evaluation of the investigators. Consequently, if at least one domain was rated as “high risk of bias,” the overall risk of bias had to be rated as “high.”

ROBINS‐I^21^ contains seven domains for assessing risk of bias: (1) bias due to confounding; (2) bias in selection of participants into the study; (3) bias in classification of interventions; (4) bias due to deviations from intended interventions; (5) bias due to missing data; (6) bias in measurement of outcomes, and (7) bias in selection of the reported result. Unlike the RoB 2 tool, the overall risk of bias of a study outcome, according to ROBINS‐I, can be classified as “low,” “moderate,” “serious,” or “critical risk of bias.”

### 2.5 Meta‐analysis

Based on data extraction, only two of the selected studies^22^, ^23^ presented suitable data regarding POS to perform a meta‐analysis. Conversely, meta‐analysis was not possible for any other clinical evaluation parameter due to significant differences in the way each study reported their results or lack of data for certain outcomes. Therefore, POS data were dichotomized as “success” or “failure,” according to the criteria used by each of the selected studies. The difference in POS between indirect restorations placed using IDS or indirect restorations placed using DDS at 1 week, 1 year, and at the final follow‐up (2 years for Hu and Zhu,^22^ and 3 years for van den Breemer et al.,^23^) was analyzed by the Revman 5.3 Software (Review Manager v. 5, The Cochrane Collaboration, Copenhagen, Denmark). The prevalence of success and the total number of restorations for each group (IDS or DDS) were used to calculate the risk difference (RD) and standard mean difference (SMD) at a confidence interval of 95%. Random‐effects models were applied, and heterogeneity was tested using the I^2^ index.

### 2.6 Certainty of evidence assessment

Quality of evidence (certainty in the estimates of effect) was determined for the POS outcome using the grading of recommendations assessment, development and evaluation (GRADE) approach^24^ which states randomized controlled clinical trials are initially considered as high‐certainty evidence, but the certainty of the body of evidence might decrease to moderate, low, or very low if serious or very serious issues concerning risk of bias, inconsistency, indirectness, imprecision, and publication bias are found. Conversely, nonrandomized studies are initially considered as low‐certainty evidence, and can be further downgraded if there are serious concerns in the five categories described above, or it may be upgraded if the magnitude of effect is large or very large or if the effect of all confounding factors would be to reduce or suggest a false effect.^25^ However, if the ROBINS‐I tool is used to assess risk of bias in nonrandomized, the evidence from nonrandomized studies is treated in the same way as that from RCTs, starting from high certainty and downgrading for any serious concerns with risk of bias, inconsistency, indirectness, imprecision, and publication bias. This is because the use of ROBINS‐I in GRADE assessments allows for a better comparison of evidence from randomized controlled trials (RCTs) and nonrandomized studies (NRSs) because they are placed on a common metric for risk of bias.^26^

## 3 RESULTS

### 3.1 Study selection

**FIGURE 1:**
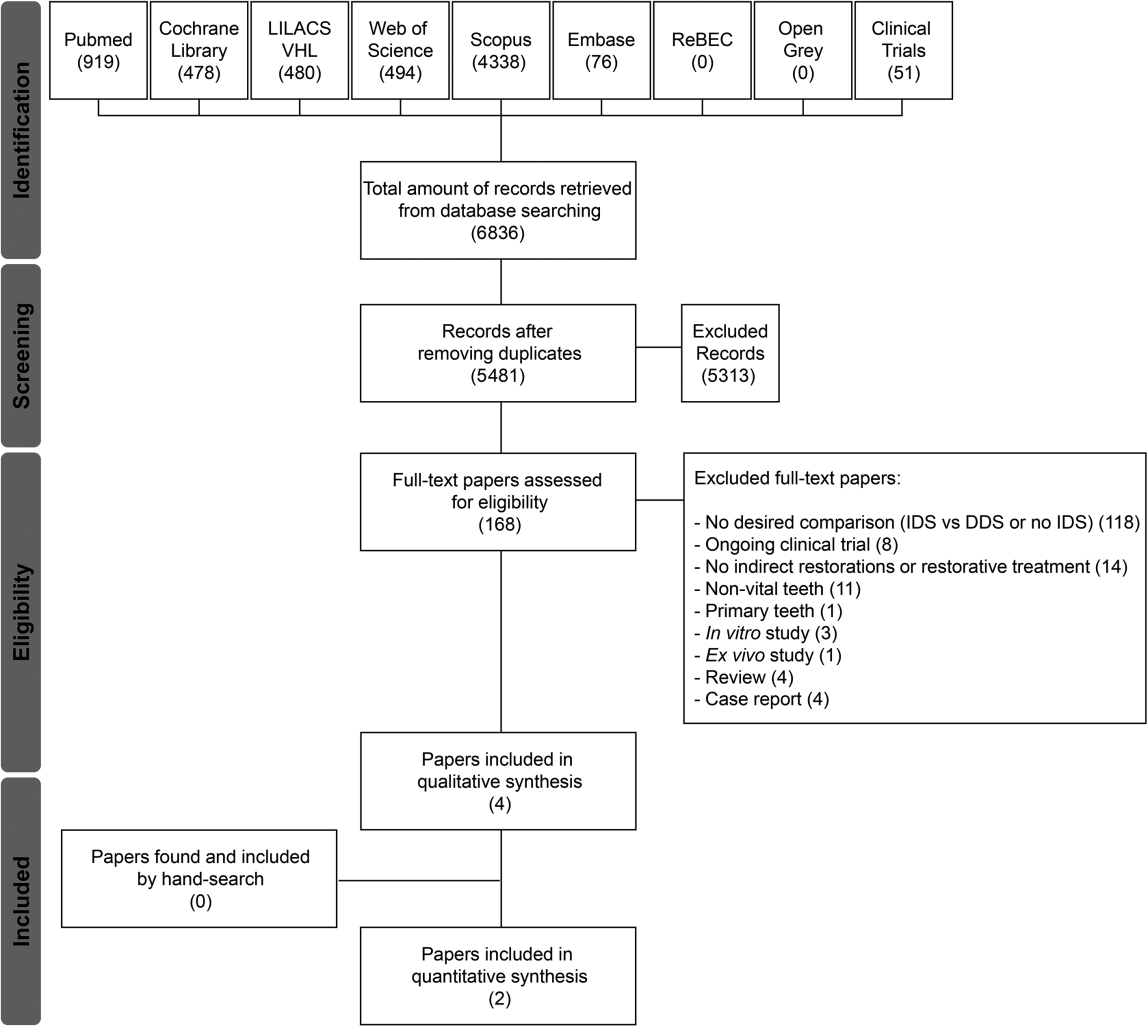
Flow diagram of study identifications

A total of 6.094 records were retrieved from the electronic search on all databases. Six hundred and thirteen duplicates were removed, leaving 5.481 papers that were screened by title and abstract. The screening process led to the exclusion of 5.316 papers that were deemed irrelevant by the investigators. Then, eligibility criteria were applied to analyze the remaining 165 papers. One hundred and seventeen papers that did not present the desired comparison (immediate vs. delayed dentin sealing), 8 ongoing clinical trials, 14 papers with no indirect restorations or restorative treatments, 11 papers evaluating non‐vital teeth, one paper evaluating primary teeth, two in vitro studies, one ex vivo study, three reviews, and four case reports were excluded. Hence, four studies were left for the systematic review, while two of these were used for meta‐analysis (Figure 1).^22^, ^23^ Additionally, it is important to highlight that manual search returned no relevant papers.

### 3.2 Descriptive analysis of the selected studies

Information regarding the four studies selected for this systematic review is shown in Table S2 (supplementary material). Out of these four studies, three were RCTs,^22^, ^23^, ^27^ and one was a prospective clinical trial (PCT).^28^ The studies were carried out in The Netherlands and China, and they were published between the years of 2010 and 2019, including a total of 554 restorations performed in 173 patients, with follow‐up periods from 1 week to 132 months. One study evaluated dental restorations by the modified United States Public Health Service (USPHS) criteria;^28^ another study used a customized sensitivity discomfort interval scale;^22^ one study applied an objective tooth sensitivity measurement coupled with a subjective tooth evaluation by the VAS scale;^27^ and one last study used the FDI criteria.^23^ All studies compared indirect restorations placed using the IDS technique with at least one other group that used the DDS technique. Also, considering the evaluation of the selected studies, the papers from van den Breemer et al.^27^ and van den Breemer et al.^23^ are both publications derived from the same cohort of patients. Therefore, in the RCTs from van den Breemer et al.,^23^, ^27^ the IDS technique was performed for 30 teeth, and the DDS approach was taken for the same number of teeth. For the PCT study,^28^ according to its published abstract, IDS was done on 87 teeth that had more than 50% of dentin exposure during restorative procedures, while 297 teeth received ceramic laminate veneers with the DDS approach, although these numbers were not clearly confirmed/stated throughout the paper. Finally, the RCT from Hu and Zhu^22^ applied IDS for 25 restorations, while other 25 restorations were performed with the DDS technique. When performing the IDS procedure, 3‐step etch‐and‐rinse,^28^ 2‐step etch‐and rinse,^22^ or 2‐step self‐etch^23^, ^27^ adhesive systems were used in the selected trials (Table S2). Additionally, in the studies from van den Breemer et al., a layer of flowable composite was applied immediately IDS, and before taking the impression for indirect restoration.^23^, ^27^ In the other studies;^22^, ^28^ however, the impression for indirect restoration was taken immediately after IDS had been performed.

### 3.3 Risk of bias of the included studies

**FIGURE 2:**
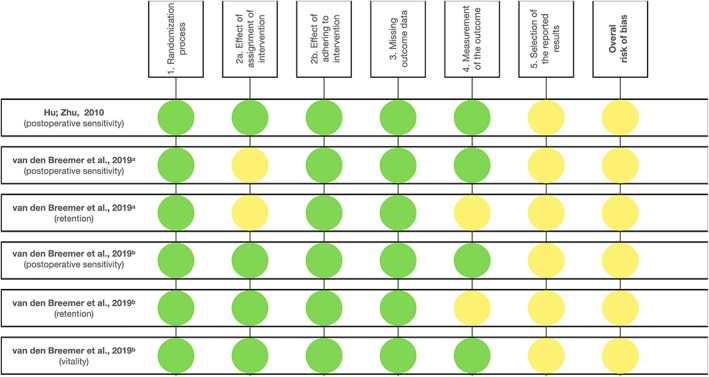
Risk of bias summary: review authors' judgments about each risk of bias item for each included study according to RoB 2 tool

**FIGURE 3:**
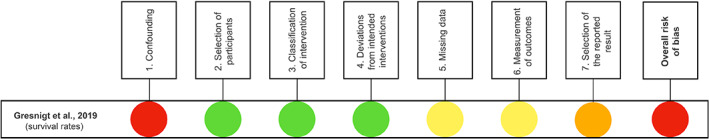
Risk of bias summary: review authors' judgments about each risk of bias item for each included study according to ROBINS‐I tool

Figures 2 and 3 summarize the results of the risk of bias analysis for the studies included in this systematic review. All studies assessed by the RoB 2 tool^22^, ^23^, ^27^ were classified as “some concerns” for domain #5 (risk of bias in selection of the reported result), because no information about whether data had been analyzed in accordance with a prespecified analysis plan was available.^20^ Consequently, the overall risk of bias for the outcome “POS” in the study from Hu and Zhu^22^ was ranked as “some concerns.”

Regarding the study by van den Breemer et al.,^27^ the overall risk of bias for POS and retention was considered as “some concerns,” since for both outcomes domain #2 revealed a lack of clarity about potential deviations from the intended intervention that could have arisen because of the trial context. Domain #4 was also problematic for this clinical trial when analyzing the outcome “retention,” as it was not clearly stated whether the assessor evaluating the outcome retention was aware of the previous intervention.

As for van den Breemer et al.,^23^ POS, retention, and vitality led to an overall risk of bias classification as “some concerns.” In the case of POS and vitality, the overall risk of bias of some concerns was caused by domain #5, as explained above. For retention, there were also concerns regarding domain #4, which contributed to its overall risk of bias.

According to the ROBINS‐I tool, the overall risk of bias for event‐free survival rates of laminate veneers reported by Gresnigt et al.^28^ was classified as “critical risk of bias” due to confounding (domain 1), which showed the percentage of dentin exposure affected the decision to use IDS, except in the earlier cohort during the first 4 years of the trial (IDS was not applied at that time). Furthermore, 14 patients were lost during follow‐up periods, and a prediction about whether these patients would have biased the specific comparison is difficult, resulting in a “moderate” risk of bias judgment for missing data (domain 5). Risk of bias in the selection of the reported result (domain 7) was judged as “serious,” as although “survival of veneers” was a planned outcome for the study as a whole, the specific result for IDS versus non‐IDS subgroup of patients may have been reported because of the significant *p* value.

### 3.4 Meta‐analysis and certainty of evidence

**FIGURE 4:**
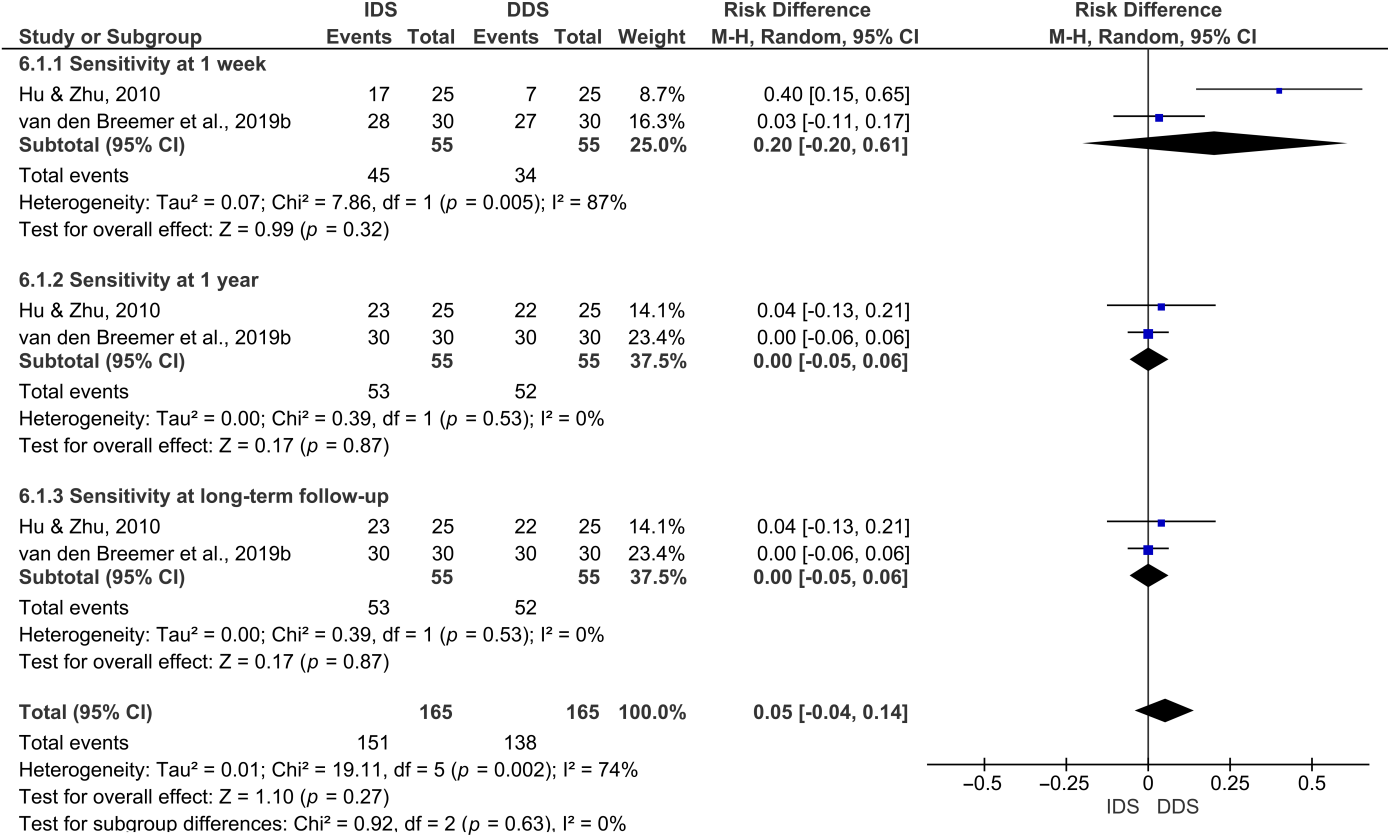
Forest plot of the postoperative sensitivity

One meta‐analysis (Figure 4) was performed for the POS parameter, taking into account the assessed follow‐up periods (one week, one year, and long‐term follow‐ups). Data for the evaluation at each follow‐up period derived from two studies.^22^, ^23^

The SMD of each follow‐up period for POS was SMD: 0.05 (−0.04, 0.14) (*p* = 0.27), whereas it was SMD: 0.20 (−0.20, 0.61) (*p* = 0.32) for POS at 1 week; SMD: 0.00 (−0.05, 0.06) (*p* = 0.87) for POS at 1 year; and SMD: 0.00 (−0.05, 0.06) (*p* = 0.87) for POS at long‐term. Moreover, the overall heterogeneity between studies was substantial (I^2^ = 74%),^29^ and for each follow‐up the heterogeneity ranged from very low (0% at 1 week and 1 year) to substantial (87% at long‐term).

Regarding the certainty of evidence, which was assessed by the GRADE tool,^29^ very low certainty of evidence was observed for POS at 1 week, with serious inconsistency and very serious imprecision. However, for POS at 1 year and at the long‐term follow‐up periods, low certainty of evidence was observed, with very serious imprecision (data shown in Table 1).

**TABLE 1:** GRADE assessment for postoperative sensitivity

Certainty assessment							No of patients		Effect		Certainty
No of studies	Study design	Risk of bias	Inconsistency	Indirectness	Imprecision	Other considerations	IDS	DDS	Relative (95% CI)	Absolute (95% CI)	
Sensitivity at 1 week											
2	Randomized trials	Not serious	Serious^a^	Not serious	Very serious^b^	None	45/55 (81.8%)	34/55 (61.8%)	RR 0.20 (−0.20 to 0.61)	495 fewer per 1.000 (from 742 fewer to 241 fewer)	⊕◯◯◯ Very low
Sensitivity at 1 year											
2	Randomized trials	Not serious	Not serious	Not serious	Very serious^b^	None	53/55 (96.4%)	52/55 (94.5%)	RR 0.00 (−0.05 to 0.06)	–per 1.000 (from 993 fewer to 889 fewer)	⊕⊕◯◯ Low
Sensitivity at long‐term follow‐up											
2	Randomized trials	Not serious	Not serious	Not serious	Very serious^b^	None	53/55 (96.4%)	52/55 (94.5%)	RR 0.00 (−0.05 to 0.06)	–per 1.000 (from 993 fewer to 889 fewer)	⊕⊕◯◯ Low

Abbreviations: CI, confidence interval; RR, risk ratio.

^a^ Heterogeneity considered substantial.

^b^ There are very few events and large confidence intervals.

## 4 DISCUSSION

Systematic reviews and meta‐analyses are at the top of the pyramid in the hierarchy of evidence in medical research.^30^ They provide useful information for clinicians during decision‐making, since they gather and summarize the knowledge about the effect of a certain treatment in a research field.^31^ A great advantage of a systematic review over a narrative review is its transparency and reduced risk of bias.^32^ Several narrative reviews that include in vitro studies concerning IDS can be found in the literature.^11^, ^14^, ^33^ However, to the best of our knowledge, systematic reviews that discuss the influence and potential benefits of the IDS technique in a clinical setting have not been published yet. Therefore, by conducting a systematic review coupled and meta‐analysis, we sought to answer whether IDS would influence POS in teeth restored with indirect restorations.

The results of the meta‐analysis revealed that IDS did not influence POS in the included clinical trials. Our meta‐analysis showed that IDS did not affect POS at 1‐week, 1‐year, or at the end of the maximum follow‐up periods available (2 and 3 years, respectively).^22^, ^23^ The two studies included in the meta‐analysis evaluated the influence of IDS in posterior teeth, which received indirect restorations both in control (DDS) and experimental (IDS) groups.^22^, ^23^ Even though differences in the choice of adhesive strategy and luting agent type could be observed between the studies, an effect of these factors on the assessed outcome is unlikely. First, Hu et al.^22^ used a 2‐step etch‐and‐rinse adhesive system, while van den Breemer et al.^23^ used a 2‐step self‐etch adhesive system (data presented in Table S2). The main difference between these strategies lies in the separate phosphoric acid‐etching step, which is performed with etch‐and‐rinse systems, while self‐etch systems do not require this separate step.^34^ It is reasonable to assume that self‐etch systems may lead to lower incidence of post‐operative sensitivity due to their less technique‐sensitive nature.^35^, ^36^ However, a recent systematic review and meta‐analysis concluded that the type of adhesive strategy (self‐etch or etch‐and‐rinse) does not influence POS of resin composite restorations in posterior teeth.^37^ Similarly, different luting agents used during cementation procedures in the two studies were not likely to influence the POS.^7^ Therefore, applying a layer of dentin bonding agent immediately after tooth preparation, that is, the IDS technique itself, regardless of the choice of the adhesive system, might have been the sole mechanism responsible for the incidence of POS in the analyzed clinical trials.

Randomized clinical trials are regarded as the optimal type of clinical research to estimate the effectiveness of a treatment intervention in oral health.^38^ Blinding is an important aspect of every randomized clinical trial and it can be applied at different levels within a study protocol, as participants, outcome assessors, care providers, or other personnel that can be blinded. Therefore, clinical trials may be defined as single‐, double‐, or triple‐blinded studies. The use of these terms; however, has not always been consistent among researchers. Nevertheless, when it is properly applied, blinding can reduce bias in research.^39^ The possibility of implementing blinding during a clinical trial depends on the assessed outcome (whether it is objective or subjective), and the type of intervention (surgical or drug delivery). In general, surgical interventions are more difficult to blind, while randomized clinical trials investigating the effect of a drug are less challenging, since placebo medications can be used as a way to facilitate the blinding process.^40^ Blinding of the operators in studies, which compared IDS against DDS was not possible due to the nature of the clinical procedure: for those teeth that underwent IDS (experimental), the layer of dentin bonding agent was applied immediately after tooth preparation and it was then light‐cured. Meanwhile, this clinical step was not implemented for teeth that were treated by the DDS technique (control). Consequently, the operators involved in these clinical procedures had to be aware of which group each tooth was assigned to, while blinding of patients could still be achieved throughout the studies.^22^, ^23^, ^27^ According to RoB 2 tool, blinding of patients has allowed the domain (5) to be classified as “low risk of bias” for the outcome POS. It is interesting to mention that, different than other clinical parameters, the assessor for the occurrence of POS was the subject itself (patient), who reported the potential sensitivity that took place after cementation. The same parallel can be drawn for the evaluation of tooth vitality, as it was evaluated based on patients' responses to cold stimuli.^23^

This systematic review also aimed to discuss the clinical behavior of indirect restorations placed with either IDS or DDS approaches. Due to different study designs^23^, ^27^, ^28^ and heterogeneity of data, a meta‐analysis for retention rates of indirect restorations was not possible. However, Van den Breemer et al. observed the same cohort of patients and published the results for 1‐year^27^ and 3‐year^23^ follow‐ups. For both dentin sealing techniques, 100% retention rates were observed at 1 year, while the overall survival rates at the 3‐year follow‐up period was 100% and 96.7% for IDS and DDS, respectively. The authors concluded there was no significant difference in survival rates of indirect restorations placed with either IDS or DDS techniques.

The rationale behind using the IDS technique during cementation is supported by its supposed beneficial effect on preventing the collapse of collagen fibers within the hybrid layer, which might occur when applying pressure while placing an indirect restoration.^11^ Consequently, an influence of IDS on retention, marginal integrity, and marginal discoloration can be expected, since these clinical parameters largely depend on the application of the adhesive system.^41^, ^42^ On the other hand, surface luster and occlusal contour wear depend on the mechanical properties of the restorative material used, and they are likely not influenced by the IDS technique.^43^, ^44^ Interestingly, no differences between IDS (86.7%) and DDS (83.3%) groups were observed after 3 years of follow‐up in the overall FDI success rate, which assessed the abovementioned clinical parameters.^23^

Finally, in a PCT, Gresnigt et al. evaluated the performance of ceramic laminate veneers placed with or without IDS, depending on the percentage of dentin exposure during preparation procedures. IDS was reported to have a beneficial effect for survival rates of laminate veneers on teeth that had more than 50% of dentin exposure, while it did not seem to influence the survival rates of teeth that had less than 50% of dentin exposure. However, the results from the study should be interpreted with caution, as it was classified as “critical risk of bias,” as previously explained (Figure 3).

Due to the small number of articles available in the literature regarding the subject of dentin sealing techniques in dentistry, the present systematic review included all clinical trials that compared the differences between IDS and DDS, irrespective of their study design. One of the potential limitations of this review is the small number of studies included in qualitative^22^, ^23^, ^27^, ^28^ and quantitative analyzes.^22^, ^23^ Our findings demonstrate that there was no statistically significant difference in the occurrence of POS when comparing IDS and DDS techniques. Although only two studies were included in the meta‐analysis, the fact that both of them showed low quality of evidence by the GRADE tool should be mentioned.^45^ However, meta‐analysis for other important clinical parameters could not be performed because of the apparent lack of studies. The quality and number of papers included in this systematic review clearly emphasize the need to conduct further randomized clinical trials with larger sample sizes and longer follow‐up periods in order to investigate the effect of IDS on esthetic, functional, and biologic properties of indirect restorations placed with this approach.

## 5 CONCLUSIONS

Within the limitations of this systematic review, it was concluded that there is low‐certainty evidence that IDS does not reduce POS in teeth restored with indirect restorations.

## DISCLOSURE

The authors do not have any financial interest in the companies whose materials are included in this article.

## Supporting information


**Table S1** Electronic databases and search strategies used in the present systematic review and meta‐analysis

**Table S2** Information about the studies included in this systematic review and meta‐analysis

## Data Availability

Data sharing not applicable to this article as no datasets were generated or analysed during the current study.
