# Supplementary material for: Does immediate dentin sealing influence postoperative sensitivity in teeth restored with indirect restorations? A systematic review and meta‐analysis
Source: J Esthet Restor Dent. 2021 Dec 3;34(1):55–64. doi: 10.1111/jerd.12841 (PMC9300029; doi:10.1111/jerd.12841)
Supplement: Supplementary file 1 — Table S1 Electronic databases and search strategies used in the present systematic review and meta‐analysis [file JERD-34-55-s002.docx]

Table 1. Electronic databases and search strategies used in the present systematic review and meta-analysis.

| Database (number of records retrieved) | Search strategy |
| --- | --- |
| Pubmed (919) | (((Crowns[MeSH Terms] OR Dental Prosthesis Retention[MeSH Terms] OR Dental Prosthesis[MeSH Terms] OR Dental Restoration Failure[MeSH Terms] OR Dental Restoration Permanent[MeSH Terms] OR Inlays[MeSH Terms] OR Dental Restoration Temporary[MeSH Terms] OR Dental Veneers[MeSH Terms] OR Denture, Partial[MeSH Terms] OR Denture, Partial, Fixed[MeSH Terms] OR Denture, Partial, Fixed, Resin-Bonded[MeSH Terms] OR Denture, Partial Immediate[MeSH Terms] OR Denture, Partial, Temporary[MeSH Terms]) OR (Crowns[Title/Abstract] OR Dental Prosthesis Retention[Title/Abstract] OR Dental Prosthesis[Title/Abstract] OR Dental Restoration Failure[Title/Abstract] OR Dental Restoration, Permanent[Title/Abstract] OR Inlays[Title/Abstract] OR Dental Restoration, Temporary[Title/Abstract] OR Dental veneers[Title/Abstract] OR Denture, Partial[Title/Abstract] OR Denture, Partial, Fixed[Title/Abstract] OR Denture, Partial, Fixed, Resin-Bonded[Title/Abstract] OR Denture, Partial, Immediate[Title/Abstract] OR Denture, Partial, Temporary[Title/Abstract] OR Crown[Title/Abstract] OR Dental Crowns[Title/Abstract] OR Dental Crown[Title/Abstract] OR Crown, Dental[Title/Abstract] OR Crowns, Dental[Title/Abstract] OR Prosthetic Crown[Title/Abstract] OR Single Crown[Title/Abstract] OR Prosthesis Retention, Dental[Title/Abstract] OR Retention, Dental Prosthesis[Title/Abstract] OR Prosthesis, Dental[Title/Abstract] OR Dental Prostheses[Title/Abstract] OR Prostheses, Dental[Title/Abstract] OR Dental Prosthodontics[Title/Abstract] OR Tooth Prosthesis[Title/Abstract] OR Tooth Prostheses[Title/Abstract] OR Tooth Prosthodontics[Title/Abstract] OR Teeth Prosthesis[Title/Abstract] OR Teeth Prostheses[Title/Abstract] OR Teeth Prosthodontics[Title/Abstract] OR Failure, Dental Restoration[Title/Abstract] OR Restoration Failure, Dental[Title/Abstract] OR Restoration Failures, Dental[Title/Abstract] OR Dental Restoration Failures[Title/Abstract] OR Failures, Dental Restoration[Title/Abstract] OR Dental Prosthesis Failure[Title/Abstract] OR Failure, Dental Prosthesis[Title/Abstract] OR Prosthesis Failure, Dental[Title/Abstract] OR Prosthesis Failures, Dental[Title/Abstract] OR Dental Prosthesis Failures[Title/Abstract] OR Failures, Dental Prosthesis[Title/Abstract] OR Restorations, Permanent Dental[Title/Abstract] OR Permanent Dental Restorations[Title/Abstract] OR Restoration, Permanent Dental[Title/Abstract] OR Dental Restorations, Permanent[Title/Abstract] OR Permanent Dental Restoration[Title/Abstract] OR Dental Permanent Fillings[Title/Abstract] OR Fillings, Permanent Dental[Title/Abstract] OR Permanent Dental Fillings[Title/Abstract] OR Permanent Fillings, Dental[Title/Abstract] OR Permanent Filling, Dental[Title/Abstract] OR Dental Filling, Permanent[Title/Abstract] OR Dental Permanent Filling[Title/Abstract] OR Filling, Dental Permanent[Title/Abstract] OR Filling, Permanent Dental[Title/Abstract] OR Permanent Dental Filling[Title/Abstract] OR Fillings, Dental Permanent[Title/Abstract] OR Dental Fillings, Permanent[Title/Abstract] OR Inlay[Title/Abstract] OR Inlay, Dental[Title/Abstract] OR Inlays, Dental[Title/Abstract] OR Dental Inlays[Title/Abstract] OR Dental Onlays[Title/Abstract] OR Dental Inlay[Title/Abstract] OR Dental Onlay[Title/Abstract] OR Onlay, Dental[Title/Abstract] OR Onlays, Dental[Title/Abstract] OR Onlays[Title/Abstract] OR Onlay[Title/Abstract] OR Tooth Inlay[Title/Abstract] OR Tooth Inlays[Title/Abstract] OR Tooth Onlay[Title/Abstract] OR Tooth Onlays[Title/Abstract] OR Overlay[Title/Abstract] OR Overlays[Title/Abstract] OR Dental Overlay[Title/Abstract] OR Dental Overlays[Title/Abstract] OR Tooth Overlay[Title/Abstract] OR Tooth Overlays[Title/Abstract] OR Temporary Dental Restorations[Title/Abstract] OR Restorations, Temporary Dental[Title/Abstract] OR Temporary Dental Restoration[Title/Abstract] OR Dental Restorations, Temporary[Title/Abstract] OR Restoration, Temporary Dental[Title/Abstract] OR Dental Prosthesis, Temporary[Title/Abstract] OR Dental Prostheses, Temporary[Title/Abstract] OR Prosthesis, Temporary Dental[Title/Abstract] OR Temporary Dental Prostheses[Title/Abstract] OR Temporary Dental Prosthesis[Title/Abstract] OR Dental Fillings, Temporary[Title/Abstract] OR Temporary Dental Fillings[Title/Abstract] OR Temporary Dental Filling[Title/Abstract] OR Dental Filling, Temporary[Title/Abstract] OR Filling, Temporary Dental[Title/Abstract] OR Fillings, Temporary Dental[Title/Abstract] OR Temporary Crown[Title/Abstract] OR Temporary Crowns[Title/Abstract] OR Veneer, Dental[Title/Abstract] OR Veneers, Dental[Title/Abstract] OR Dental Laminates[Title/Abstract] OR Dental Laminate[Title/Abstract] OR Laminate, Dental[Title/Abstract] OR Laminates, Dental[Title/Abstract] OR Dental Veneer[Title/Abstract] OR Ceramic Laminate[Title/Abstract] OR Ceramic Laminates[Title/Abstract] OR Porcelain Laminate[Title/Abstract] OR Porcelain Laminates[Title/Abstract] OR Dental Contact Lenses[Title/Abstract] OR Dental Contact Lens[Title/Abstract] OR Contact Lens[Title/Abstract] OR Contact Lenses[Title/Abstract] OR Dentures, Partial[Title/Abstract] OR Partial Denture[Title/Abstract] OR Partial Dentures[Title/Abstract] OR Bridgework, Dental[Title/Abstract] OR Bridgeworks, Dental[Title/Abstract] OR Dental Bridgeworks[Title/Abstract] OR Dental Bridgework[Title/Abstract] OR Fixed Bridge[Title/Abstract] OR Bridge, Fixed[Title/Abstract] OR Bridges, Fixed[Title/Abstract] OR Fixed Bridges[Title/Abstract] OR Fixed Partial Denture[Title/Abstract] OR Denture, Fixed Partial[Title/Abstract] OR Dentures, Fixed Partial[Title/Abstract] OR Fixed Partial Dentures[Title/Abstract] OR Partial Denture, Fixed[Title/Abstract] OR Partial Dentures, Fixed[Title/Abstract] OR Pontic[Title/Abstract] OR Pontics[Title/Abstract] OR Resin-Bonded Bridge[Title/Abstract] OR Bridge, Resin-Bonded[Title/Abstract] OR Bridges, Resin-Bonded[Title/Abstract] OR Resin Bonded Bridge[Title/Abstract] OR Resin-Bonded Bridges[Title/Abstract] OR Resin-Bonded Acid-Etched Fixed Partial Denture[Title/Abstract] OR Resin Bonded Acid Etched Fixed Partial Denture[Title/Abstract] OR Maryland Bridge[Title/Abstract] OR Bridge, Maryland[Title/Abstract] OR Resin-Bonded Fixed Partial Denture[Title/Abstract] OR Resin Bonded Fixed Partial Denture[Title/Abstract] OR Interim Dental Prosthesis[Title/Abstract] OR Dental Prostheses, Interim[Title/Abstract] OR Interim Dental Prostheses[Title/Abstract] OR Prostheses, Interim Dental[Title/Abstract] OR Prosthesis, Interim Dental[Title/Abstract] OR Dental Prosthesis, Interim[Title/Abstract] OR Interim Prosthesis Dental[Title/Abstract] OR Dental, Interim Prosthesis[Title/Abstract] OR Dentals, Interim Prosthesis[Title/Abstract] OR Interim Prosthesis Dentals[Title/Abstract] OR Prosthesis Dental, Interim[Title/Abstract] OR Prosthesis Dentals, Interim[Title/Abstract])) AND ((Dentin Desensitizing Agents[MeSH Terms] OR Dentin Sensitivity[MeSH Terms] OR Dentin Permeability[MeSH Terms] OR Tooth Permeability[MeSH Terms] OR Dental Enamel Permeability[MeSH Terms]) OR (Dentin Desensitizing Agents[Title/Abstract] OR Dentin Sensitivity[Title/Abstract] OR Dentin Permeability[Title/Abstract] OR Tooth Permeability[Title/Abstract] OR Dental Enamel Permeability[Title/Abstract] OR Agents, Dentin Desensitizing[Title/Abstract] OR Desensitizing Agents, Dentin[Title/Abstract] OR Dentine Desensitizing Agents[Title/Abstract] OR Agents, Dentine Desensitizing[Title/Abstract] OR Desensitizing Agents, Dentine[Title/Abstract] OR Dentin Desensitizer[Title/Abstract] OR Dentin Desensitizers[Title/Abstract] OR Dentine Desensitizer[Title/Abstract] OR Dentine Desensitizers[Title/Abstract] OR Dentin Sensitivities[Title/Abstract] OR Sensitivities, Dentin[Title/Abstract] OR Sensitivity, Dentin[Title/Abstract] OR Dentine Hypersensitivity[Title/Abstract] OR Dentine Hypersensitivities[Title/Abstract] OR Hypersensitivities, Dentine[Title/Abstract] OR Hypersensitivity, Dentine[Title/Abstract] OR Dentine Sensitivity[Title/Abstract] OR Dentine Sensitivities[Title/Abstract] OR Sensitivities, Dentine[Title/Abstract] OR Sensitivity, Dentine[Title/Abstract] OR Tooth Sensitivity[Title/Abstract] OR Sensitivities, Tooth[Title/Abstract] OR Sensitivity, Tooth[Title/Abstract] OR Tooth Sensitivities[Title/Abstract] OR Dentin Hypersensitivity[Title/Abstract] OR Dentin Hypersensitivities[Title/Abstract] OR Hypersensitivities, Dentin[Title/Abstract] OR Hypersensitivity, Dentin[Title/Abstract] OR Dentin Permeabilities[Title/Abstract] OR Permeabilities, Dentin[Title/Abstract] OR Permeability, Dentin[Title/Abstract] OR Dentine Permeability[Title/Abstract] OR Dentine Permeabilities[Title/Abstract] OR Permeabilities, Dentine[Title/Abstract] OR Permeability, Dentine[Title/Abstract] OR Permeability, Tooth[Title/Abstract] OR Permeabilities, Tooth[Title/Abstract] OR Tooth Permeabilities[Title/Abstract] OR Permeability, Dental Enamel[Title/Abstract] OR Enamel Permeability, Dental[Title/Abstract] OR Dentin immediate sealing[Title/Abstract] OR Immediate dentin sealing[Title/Abstract] OR Dentine immediate sealing[Title/Abstract] OR Immediate dentine sealing[Title/Abstract] OR Dentin sealing[Title/Abstract] OR Dentine sealing[Title/Abstract] OR Dentin seal[Title/Abstract] OR Dentine seal[Title/Abstract] OR IDS[Title/Abstract] OR Pre-hybridization[Title/Abstract] OR Pre hybridization[Title/Abstract] OR Prehybridization[Title/Abstract] OR Pre-hybridisation[Title/Abstract] OR Pre hybridization[Title/Abstract] OR Prehybridisation[Title/Abstract] OR resin coating[Title/Abstract] OR resin-coating[Title/Abstract]))) AND (Clinical[Title/Abstract] OR Randomized[Title/Abstract] OR Intervention Study[Title/Abstract] OR Intervention Studies[Title/Abstract] OR Controlled Trial*[Title/Abstract] OR Prospective[Title/Abstract] OR Follow-up*[Title/Abstract] OR follow up[Title/Abstract] OR Trial*[Title/Abstract] OR Longitudinal[Title/Abstract] OR Quasi-Experimental[Title/Abstract] OR Non-Randomized[Title/Abstract] OR Nonrandomized[Title/Abstract]) |
| Cochrane Library (478) | 1- MeSH descriptor: [Crowns] explode all trees  2- (Crown OR Dental Crowns OR Dental Crown OR Crown, Dental OR Crowns, Dental OR Prosthetic Crown OR Single Crown):ti,ab,kw  3- MeSH descriptor: [Dental Prosthesis Retention] explode all trees  4- (Prosthesis Retention, Dental OR Retention, Dental Prosthesis):ti,ab,kw  5- MeSH descriptor: [Dental Prosthesis] explode all trees  6- (Prosthesis, Dental OR Dental Prostheses OR Prostheses, Dental OR Dental Prosthodontics OR Tooth Prosthesis OR Tooth Prostheses OR Tooth Prosthodontics OR Teeth Prosthesis OR Teeth Prostheses OR Teeth Prosthodontics):ti,ab,kw  7- MeSH descriptor: [Dental Restoration Failure] explode all trees  8- (Failure, Dental Restoration OR Restoration Failure, Dental OR Restoration Failures, Dental OR Dental Restoration Failures OR Failures, Dental Restoration OR Dental Prosthesis Failure OR Failure, Dental Prosthesis OR Prosthesis Failure, Dental OR Prosthesis Failures, Dental OR Dental Prosthesis Failures OR Failures, Dental Prosthesis):ti,ab,kw  9- MeSH descriptor: [Dental Restoration, Permanent] explode all trees  10- (Restorations, Permanent Dental OR Permanent Dental Restorations OR Restoration, Permanent Dental OR Dental Restorations, Permanent OR Permanent Dental Restoration OR Dental Permanent Fillings OR Fillings, Permanent Dental OR Permanent Dental Fillings OR Permanent Fillings, Dental OR Permanent Filling, Dental OR Dental Filling, Permanent OR Dental Permanent Filling OR Filling, Dental Permanent OR Filling, Permanent Dental OR Permanent Dental Filling OR Fillings, Dental Permanent OR Dental Fillings, Permanent):ti,ab,kw  11- MeSH descriptor: [Inlays] explode all trees  12- (Inlay OR Inlay, Dental OR Inlays, Dental OR Dental Inlays OR Dental Onlays OR Dental Inlay OR Dental Onlay OR Onlay, Dental OR Onlays, Dental OR Onlays OR Onlay):ti,ab,kw  13- (Tooth Inlay OR Tooth Inlays OR Tooth Onlay OR Tooth Onlays OR Overlay OR Overlays OR Dental Overlay OR Dental Overlays OR Tooth Overlay OR Tooth Overlays):ti,ab,kw  14- MeSH descriptor: [Dental Restoration, Temporary] explode all trees  15- (Temporary Dental Restorations OR Restorations, Temporary Dental OR Temporary Dental Restoration OR Dental Restorations, Temporary OR Restoration, Temporary Dental OR Dental Prosthesis, Temporary OR Dental Prostheses, Temporary OR Prosthesis, Temporary Dental OR Temporary Dental Prostheses OR Temporary Dental Prosthesis OR Dental Fillings, Temporary OR Temporary Dental Fillings OR Temporary Dental Filling OR Dental Filling, Temporary OR Filling, Temporary Dental OR Fillings, Temporary Dental OR Temporary Crown OR Temporary Crowns):ti,ab,kw  16- MeSH descriptor: [Dental Veneers] explode all trees  17- (Veneer, Dental OR Veneers, Dental OR Dental Laminates OR Dental Laminate OR Laminate, Dental OR Laminates, Dental OR Dental Veneer OR Ceramic Laminate OR Ceramic Laminates OR Porcelain Laminate OR Porcelain Laminates OR Dental Contact Lenses OR Dental Contact Lens OR Contact Lens OR Contact Lenses):ti,ab,kw  18- MeSH descriptor: [Denture, Partial] explode all trees  19- (Dentures, Partial OR Partial Denture OR Partial Dentures OR Bridgework, Dental OR Bridgeworks, Dental OR Dental Bridgeworks OR Dental Bridgework):ti,ab,kw  20- MeSH descriptor: [Denture, Partial, Fixed] explode all trees  21- (Fixed Bridge OR Bridge, Fixed OR Bridges, Fixed OR Fixed Bridges OR Fixed Partial Denture OR Denture, Fixed Partial OR Dentures, Fixed Partial OR Fixed Partial Dentures OR Partial Denture, Fixed OR Partial Dentures, Fixed OR Pontic OR Pontics):ti,ab,kw  22- MeSH descriptor: [Denture, Partial, Fixed, Resin-Bonded] explode all trees  23- (Resin-Bonded Bridge OR Bridge, Resin-Bonded OR Bridges, Resin-Bonded OR Resin Bonded Bridge OR Resin-Bonded Bridges OR Resin-Bonded Acid-Etched Fixed Partial Denture OR Resin Bonded Acid Etched Fixed Partial Denture OR Maryland Bridge OR Bridge, Maryland OR Resin-Bonded Fixed Partial Denture OR Resin Bonded Fixed Partial Denture):ti,ab,kw  24- MeSH descriptor: [Denture, Partial, Immediate] explode all trees  25- MeSH descriptor: [Denture, Partial, Temporary] explode all trees  26- (Interim Dental Prosthesis OR Dental Prostheses, Interim OR Interim Dental Prostheses OR Prostheses, Interim Dental OR Prosthesis, Interim Dental OR Dental Prosthesis, Interim OR Interim Prosthesis Dental OR Dental, Interim Prosthesis OR Dentals, Interim Prosthesis OR Interim Prosthesis Dentals OR Prosthesis Dental, Interim OR Prosthesis Dentals, Interim):ti,ab,kw  27- #1 OR #2 OR #3 OR #4 OR #5 OR #6 OR #7 OR #8 OR #9 OR #10 OR #11 OR #12 OR #13 OR #14 OR #15 OR #16 OR #17 OR #18 OR #19 OR #20 OR #21 OR #22 OR #23 OR #24 OR #25 OR #26  28- MeSH descriptor: [Dentin Desensitizing Agents] explode all trees  29- (Agents, Dentin Desensitizing OR Desensitizing Agents, Dentin OR Dentine Desensitizing Agents OR Agents, Dentine Desensitizing OR Desensitizing Agents, Dentine OR Dentin Desensitizer OR Dentin Desensitizers OR Dentine Desensitizer OR Dentine Desensitizers):ti,ab,kw  30- MeSH descriptor: [Dentin Sensitivity] explode all trees  31- (Dentin Sensitivities OR Sensitivities, Dentin OR Sensitivity, Dentin OR Dentine Hypersensitivity OR Dentine Hypersensitivities OR Hypersensitivities, Dentine OR Hypersensitivity, Dentine OR Dentine Sensitivity OR Dentine Sensitivities OR Sensitivities, Dentine OR Sensitivity, Dentine OR Tooth Sensitivity OR Sensitivities, Tooth OR Sensitivity, Tooth OR Tooth Sensitivities OR Dentin Hypersensitivity OR Dentin Hypersensitivities OR Hypersensitivities, Dentin OR Hypersensitivity, Dentin):ti,ab,kw  32- MeSH descriptor: [Dentin Permeability] explode all trees  33- (Dentin Permeabilities OR Permeabilities, Dentin OR Permeability, Dentin OR Dentine Permeability OR Dentine Permeabilities OR Permeabilities, Dentine OR Permeability, Dentine):ti,ab,kw  34- MeSH descriptor: [Tooth Permeability] explode all trees  35- (Permeability, Tooth OR Permeabilities, Tooth OR Tooth Permeabilities):ti,ab,kw  36- MeSH descriptor: [Dental Enamel Permeability] explode all trees  37- (Permeability, Dental Enamel OR Enamel Permeability, Dental):ti,ab,kw  38- (Dentin immediate sealing OR Immediate dentin sealing OR Dentine immediate sealing OR Immediate dentine sealing OR Dentin sealing OR Dentine sealing OR Dentin seal OR Dentine seal OR IDS OR Pre-hybridization OR Pre hybridization OR Prehybridization OR Pre-hybridisation OR Pre hybridization OR Prehybridisation):ti,ab,kw  39- (Resin coating):ti,ab,kw  40- (Resin-coating):ti,ab,kw  41- #28 OR #29 OR #30 OR #31 OR #32 OR #33 OR #34 OR #35 OR #36 OR #37 OR #38 OR #39 OR #40  42- (Clinical OR Randomized OR Intervention Study OR Intervention Studies OR Controlled Trial* OR Prospective OR Follow-up* OR Follow Up OR Trial* OR Longitudinal OR Quasi-Experimental OR Non-Randomized OR Nonrandomized):ti,ab,kw  **43- #27 AND #41 AND #42** |
| Virtual Health Library (VHL) LILACS (480) | tw:((tw:((mh:(crowns)) OR (tw:(crown OR dental crowns OR dental crown OR crown, dental OR crowns, dental OR prosthetic crown OR single crown)) OR (mh:(dental prosthesis retention)) OR (tw:(prosthesis retention, dental OR retention, dental prosthesis)) OR (mh:(dental prosthesis)) OR (tw:(prosthesis, dental OR dental prostheses OR prostheses, dental OR dental prosthodontics OR tooth prosthesis OR tooth prostheses OR tooth prosthodontics OR teeth prosthesis OR teeth prostheses OR teeth prosthodontics)) OR (mh:(dental restoration failure)) OR (tw:(failure, dental restoration OR restoration failure, dental OR restoration failures, dental OR dental restoration failures OR failures, dental restoration OR dental prosthesis failure OR failure, dental prosthesis OR prosthesis failure, dental OR prosthesis failures, dental OR dental prosthesis failures OR failures, dental prosthesis)) OR (mh:(dental restoration, permanent)) OR (tw:(restorations, permanent dental OR permanent dental restorations OR restoration, permanent dental OR dental restorations, permanent OR permanent dental restoration OR dental permanent fillings OR fillings, permanent dental OR permanent dental fillings OR permanent fillings, dental OR permanent filling, dental OR dental filling, permanent OR dental permanent filling OR filling, dental permanent OR filling, permanent dental OR permanent dental filling OR fillings, dental permanent OR dental fillings, permanent)) OR (mh:(inlays)) OR (tw:(inlay OR inlay, dental OR inlays, dental OR dental inlays OR dental onlays OR dental inlay OR dental onlay OR onlay, dental OR onlays, dental OR onlays onlay)) OR (tw:(tooth inlay OR tooth inlays OR tooth onlay OR tooth onlays OR overlay OR overlays OR dental overlay OR dental overlays OR tooth overlay OR tooth overlays)) OR (mh:(dental restoration, temporary)) OR (tw:(temporary dental restorations OR restorations, temporary dental OR temporary dental restoration OR dental restorations, temporary OR restoration, temporary dental OR dental prosthesis, temporary OR dental prostheses, temporary OR prosthesis, temporary dental OR temporary dental prostheses OR temporary dental prosthesis OR dental fillings, temporary OR temporary dental fillings OR temporary dental filling OR dental filling, temporary OR filling, temporary dental OR fillings, temporary denta OR temporary crown OR temporary crownsl)) OR (mh:(dental veneers)) OR (tw:(veneer, dental OR veneers, dental OR dental laminates OR dental laminate OR laminate, dental OR laminates, dental OR dental veneer)) OR (tw:(ceramic laminate OR ceramic laminates OR porcelain laminate OR porcelain laminates OR dental contact lenses OR dental contact lens OR contact lens OR contact lenses)) OR (mh:(denture, partial)) OR (tw:(dentures, partial OR partial denture OR partial dentures OR bridgework, dental OR bridgeworks, dental OR dental bridgeworks OR dental bridgework)) OR (mh:(denture, partial, fixed)) OR (tw:(fixed bridge OR bridge, fixed OR bridges, fixed OR fixed bridges OR fixed partial denture OR denture, fixed partial OR dentures, fixed partial OR fixed partial dentures OR partial denture, fixed OR partial dentures, fixed OR pontic OR pontics)) OR (mh:(denture, partial, fixed, resin-bonded)) OR (tw:(resin-bonded bridge OR bridge, resin-bonded OR bridges, resin-bonded OR resin bonded bridge OR resin-bonded bridges OR resin-bonded acid-etched fixed partial denture OR resin bonded acid etched fixed partial denture OR maryland bridge OR bridge, maryland OR resin-bonded fixed partial denture OR resin bonded fixed partial denture)) OR (mh:(denture, partial, immediate)) OR (mh:(denture, partial, temporary)) OR (tw:(interim dental prosthesis OR dental prostheses, interim OR interim dental prostheses OR prostheses, interim dental OR prosthesis, interim dental OR dental prosthesis, interim OR interim prosthesis dental OR dental, interim prosthesis OR dentals, interim prosthesis OR interim prosthesis dentals OR prosthesis dental, interim OR prosthesis dentals, interim)))) AND (tw:((mh:(dentin desensitizing agents)) OR (tw:(agents, dentin desensitizing OR desensitizing agents, dentin)) OR (tw:(dentine desensitizing agents OR agents, dentine desensitizing OR desensitizing agents, dentine OR dentin desensitizer OR dentin desensitizers OR dentine desensitizer OR dentine desensitizers)) OR (mh:(dentin sensitivity)) OR (tw:(dentin sensitivities OR sensitivities, dentin OR sensitivity, dentin OR dentine hypersensitivity OR dentine hypersensitivities OR hypersensitivities, dentine OR hypersensitivity, dentine OR dentine sensitivity OR dentine sensitivities OR sensitivities, dentine OR sensitivity, dentine OR tooth sensitivity OR sensitivities, tooth OR sensitivity, tooth OR tooth sensitivities OR dentin hypersensitivity OR dentin hypersensitivities OR hypersensitivities, dentin OR hypersensitivity, dentin)) OR (mh:(dentin permeability)) OR (tw:(dentin permeabilities OR permeabilities, dentin OR permeability, dentin OR dentine permeability OR dentine permeabilities OR permeabilities, dentine OR permeability, dentine)) OR (mh:(tooth permeability)) OR (tw:(permeability, tooth OR permeabilities, tooth OR tooth permeabilities)) OR (mh:(dental enamel permeability)) OR (tw:(permeability, dental enamel OR enamel permeability, dental)) OR (tw:(dentin immediate sealing OR immediate dentin sealing OR dentine immediate sealing OR immediate dentine sealing OR dentin sealing OR dentine sealing OR dentin seal OR dentine seal OR ids OR pre-hybridization OR pre hybridization OR prehybridization OR pre-hybridisation OR pre hybridization OR prehybridization OR resin coating OR resin-coating)))) AND (tw:((tw:(clinical OR randomized OR intervention study OR intervention studies OR controlled trial* OR prospective OR follow-up* OR follow up OR trial* OR longitudinal OR quasi-experimental OR non-randomized OR nonrandomized))))) AND ( db:("LILACS")) |
| Web of Science (494) | **#1**  TS=(Crowns OR Crown OR Dental Crowns OR Dental Crown OR Crown, Dental OR Crowns, Dental OR Prosthetic Crown OR Single Crown OR Dental Prosthesis Retention OR Prosthesis Retention, Dental OR Retention, Dental Prosthesis OR Dental Prosthesis OR Prosthesis, Dental OR Dental Prostheses OR Prostheses, Dental OR Dental Prosthodontics OR Tooth Prosthesis OR Tooth Prostheses OR Tooth Prosthodontics OR Teeth Prosthesis OR Teeth Prostheses OR Teeth Prosthodontics OR Dental Restoration Failure OR Failure, Dental Restoration OR Restoration Failure, Dental OR Restoration Failures, Dental OR Dental Restoration Failures OR Failures, Dental Restoration OR Dental Prosthesis Failure OR Failure, Dental Prosthesis OR Prosthesis Failure, Dental OR Prosthesis Failures, Dental OR Dental Prosthesis Failures OR Failures, Dental Prosthesis OR Dental Restoration, Permanent OR Restorations, Permanent Dental OR Permanent Dental Restorations OR Restoration, Permanent Dental OR Dental Restorations, Permanent OR Permanent Dental Restoration OR Dental Permanent Fillings OR Fillings, Permanent Dental OR Permanent Dental Fillings OR Permanent Fillings, Dental OR Permanent Filling, Dental OR Dental Filling, Permanent OR Dental Permanent Filling OR Filling, Dental Permanent OR Filling, Permanent Dental OR Permanent Dental Filling OR Fillings, Dental Permanent OR Dental Fillings, Permanent OR Inlays OR Inlay OR Inlay, Dental OR Inlays, Dental OR Dental Inlays OR Dental Onlays OR Dental Inlay OR Dental Onlay OR Onlay, Dental OR Onlays, Dental OR Onlays OR Onlay OR Tooth Inlay OR Tooth Inlays OR Tooth Onlay OR Tooth Onlays OR Overlay OR Overlays OR Dental Overlay OR Dental Overlays OR Tooth Overlay OR Tooth Overlays OR Dental Restoration, Temporary OR Temporary Dental Restorations OR Restorations, Temporary Dental OR Temporary Dental Restoration OR Dental Restorations, Temporary OR Restoration, Temporary Dental OR Dental Prosthesis, Temporary OR Dental Prostheses, Temporary OR Prosthesis, Temporary Dental OR Temporary Dental Prostheses OR Temporary Dental Prosthesis OR Dental Fillings, Temporary OR Temporary Dental Fillings OR Temporary Dental Filling OR Dental Filling, Temporary OR Filling, Temporary Dental OR Fillings, Temporary Dental OR Temporary Crown OR Temporary Crowns OR Dental veneers OR Veneer, Dental OR Veneers, Dental OR Dental Laminates OR Dental Laminate OR Laminate, Dental OR Laminates, Dental OR Dental Veneer OR Ceramic Laminate OR Ceramic Laminates OR Porcelain Laminate OR Porcelain Laminates OR Dental Contact Lenses OR Dental Contact Lens OR Contact Lens OR Contact Lenses OR Denture, Partial OR Dentures, Partial OR Partial Denture OR Partial Dentures OR Bridgework, Dental OR Bridgeworks, Dental OR Dental Bridgeworks OR Dental Bridgework OR Denture, Partial, Fixed OR Fixed Bridge OR Bridge, Fixed OR Bridges, Fixed OR Fixed Bridges OR Fixed Partial Denture OR Denture, Fixed Partial OR Dentures, Fixed Partial OR Fixed Partial Dentures OR Partial Denture, Fixed OR Partial Dentures, Fixed OR Pontic OR Pontics OR Denture, Partial, Fixed, Resin-Bonded OR Resin-Bonded Bridge OR Bridge, Resin-Bonded OR Bridges, Resin-Bonded OR Resin Bonded Bridge OR Resin-Bonded Bridges OR Resin-Bonded Acid-Etched Fixed Partial Denture OR Resin Bonded Acid Etched Fixed Partial Denture OR Maryland Bridge OR Bridge, Maryland OR Resin-Bonded Fixed Partial Denture OR Resin Bonded Fixed Partial Denture OR Denture, Partial, Immediate OR Denture, Partial, Temporary OR Interim Dental Prosthesis OR Dental Prostheses, Interim OR Interim Dental Prostheses OR Prostheses, Interim Dental OR Prosthesis, Interim Dental OR Dental Prosthesis, Interim OR Interim Prosthesis Dental OR Dental, Interim Prosthesis OR Dentals, Interim Prosthesis OR Interim Prosthesis Dentals OR Prosthesis Dental, Interim OR Prosthesis Dentals, Interim)  **#2**  TS=(Dentin Desensitizing Agents OR Agents, Dentin Desensitizing OR Desensitizing Agents, Dentin OR Dentine Desensitizing Agents OR Agents, Dentine Desensitizing OR Desensitizing Agents, Dentine OR Dentin Desensitizer OR Dentin Desensitizers OR Dentine Desensitizer OR Dentine Desensitizers OR Dentin Sensitivity OR Dentin Sensitivities OR Sensitivities, Dentin OR Sensitivity, Dentin OR Dentine Hypersensitivity OR Dentine Hypersensitivities OR Hypersensitivities, Dentine OR Hypersensitivity, Dentine OR Dentine Sensitivity OR Dentine Sensitivities OR Sensitivities, Dentine OR Sensitivity, Dentine OR Tooth Sensitivity OR Sensitivities, Tooth OR Sensitivity, Tooth OR Tooth Sensitivities OR Dentin Hypersensitivity OR Dentin Hypersensitivities OR Hypersensitivities, Dentin OR Hypersensitivity, Dentin OR Dentin Permeability OR Dentin Permeabilities OR Permeabilities, Dentin OR Permeability, Dentin OR Dentine Permeability OR Dentine Permeabilities OR Permeabilities, Dentine OR Permeability, Dentine OR Tooth Permeability OR Permeability, Tooth OR Permeabilities, Tooth OR Tooth Permeabilities OR Dental Enamel Permeability OR Permeability, Dental Enamel OR Enamel Permeability, Dental OR Dentin immediate sealing OR Immediate dentin sealing OR Dentine immediate sealing OR Immediate dentine sealing OR Dentin sealing OR Dentine sealing OR Dentin seal OR Dentine seal OR IDS OR Pre-hybridization OR Pre hybridization OR Prehybridization OR Pre-hybridisation OR Pre hybridization OR Prehybridisation OR “Resin coating” OR resin-coating)  **#3**  TS=(Clinical OR Randomized OR Intervention Study OR Intervention Studies OR Controlled Trial* OR Prospective OR Follow-up* OR Follow Up OR Trial* OR Longitudinal OR Quasi-Experimental OR Non-Randomized OR Nonrandomized)  **#1 AND #2 AND #3** |
| Scopus (4,338) | (( ( ( TITLE-ABS-KEY ( crowns ) OR TITLE-ABS-KEY ( crown ) OR TITLE-ABS-KEY ( "Dental Crowns" ) OR TITLE-ABS-KEY ( "Dental Crown" ) OR TITLE-ABS-KEY ( "Crown, Dental" ) ) ) OR ( ( TITLE-ABS-KEY ( "Crowns, Dental" ) OR TITLE-ABS-KEY ( "Prosthetic Crown" ) OR TITLE-ABS-KEY ( "Single Crown" ) OR TITLE-ABS-KEY ( "Dental Prosthesis Retention" ) OR TITLE-ABS-KEY ( "Prosthesis Retention, Dental" ) OR TITLE-ABS-KEY ( "Retention, Dental Prosthesis" ) OR TITLE-ABS-KEY ( "Dental Prosthesis" ) ) ) OR ( ( TITLE-ABS-KEY ( "Prosthesis, Dental" ) OR TITLE-ABS-KEY ( "Dental Prostheses" ) OR TITLE-ABS-KEY ( "Prostheses, Dental" ) OR TITLE-ABS-KEY ( "Dental Prosthodontics" ) OR TITLE-ABS-KEY ( "Tooth Prosthesis" ) OR TITLE-ABS-KEY ( "Tooth Prostheses" ) OR TITLE-ABS-KEY ( "Tooth Prosthodontics" ) OR TITLE-ABS-KEY ( "Teeth Prosthesis" ) ) ) OR ( ( TITLE-ABS-KEY ( "Teeth Prostheses" ) OR TITLE-ABS-KEY ( "Teeth Prosthodontics" ) OR TITLE-ABS-KEY ( "Dental Restoration Failure" ) OR TITLE-ABS-KEY ( "Failure, Dental Restoration" ) OR TITLE-ABS-KEY ( "Restoration Failure, Dental" ) OR TITLE-ABS-KEY ( "Restoration Failures, Dental" ) OR TITLE-ABS-KEY ( "Dental Restoration Failures" ) OR TITLE-ABS-KEY ( "Failures, Dental Restoration" ) ) ) OR 7 OR ( ( TITLE-ABS-KEY ( "Permanent Dental Restorations" ) OR TITLE-ABS-KEY ( "Restoration, Permanent Dental" ) OR TITLE-ABS-KEY ( "Dental Restorations, Permanent" ) OR TITLE-ABS-KEY ( "Permanent Dental Restoration" ) OR TITLE-ABS-KEY ( "Dental Permanent Fillings" ) OR TITLE-ABS-KEY ( "Fillings, Permanent Dental" ) OR TITLE-ABS-KEY ( "Permanent Dental Fillings" ) OR TITLE-ABS-KEY ( "Permanent Fillings, Dental" ) ) ) OR ( ( TITLE-ABS-KEY ( "Permanent Filling, Dental" ) OR TITLE-ABS-KEY ( "Dental Filling, Permanent" ) OR TITLE-ABS-KEY ( "Dental Permanent Filling" ) OR TITLE-ABS-KEY ( "Filling, Dental Permanent" ) OR TITLE-ABS-KEY ( "Filling, Permanent Dental" ) OR TITLE-ABS-KEY ( "Permanent Dental Filling" ) OR TITLE-ABS-KEY ( "Fillings, Dental Permanent" ) OR TITLE-ABS-KEY ( "Dental Fillings, Permanent" ) ) ) OR ( ( TITLE-ABS-KEY ( inlays ) OR TITLE-ABS-KEY ( inlay ) OR TITLE-ABS-KEY ( "Inlay, Dental" ) OR TITLE-ABS-KEY ( "Inlays, Dental" ) OR TITLE-ABS-KEY ( "Dental Inlays" ) OR TITLE-ABS-KEY ( "Dental Onlays" ) OR TITLE-ABS-KEY ( "Dental Inlay" ) OR TITLE-ABS-KEY ( "Dental Onlay" ) ) ) OR ( ( TITLE-ABS-KEY ( "Onlay, Dental" ) OR TITLE-ABS-KEY ( "Onlays, Dental" ) OR TITLE-ABS-KEY ( onlays ) OR TITLE-ABS-KEY ( onlay ) OR TITLE-ABS-KEY ( "Tooth Inlay" ) OR TITLE-ABS-KEY ( "Tooth Inlays" ) OR TITLE-ABS-KEY ( "Tooth Onlay" ) OR TITLE-ABS-KEY ( "Tooth Onlays" ) ) ) OR ( ( TITLE-ABS-KEY ( overlay ) OR TITLE-ABS-KEY ( overlays ) OR TITLE-ABS-KEY ( "Dental Overlay" ) OR TITLE-ABS-KEY ( "Dental Overlays" ) OR TITLE-ABS-KEY ( "Tooth Overlay" ) OR TITLE-ABS-KEY ( "Tooth Overlays" ) OR TITLE-ABS-KEY ( "Dental Restoration, Temporary" ) OR TITLE-ABS-KEY ( "Temporary Dental Restorations" ) ) ) OR ( ( TITLE-ABS-KEY ( "Restorations, Temporary Dental" ) OR TITLE-ABS-KEY ( "Temporary Dental Restoration" ) OR TITLE-ABS-KEY ( "Dental Restorations, Temporary" ) OR TITLE-ABS-KEY ( "Restoration, Temporary Dental" ) OR TITLE-ABS-KEY ( "Dental Prosthesis, Temporary" ) OR TITLE-ABS-KEY ( "Dental Prostheses, Temporary" ) OR TITLE-ABS-KEY ( "Prosthesis, Temporary Dental" ) OR TITLE-ABS-KEY ( "Temporary Dental Prostheses" ) ) ) OR ( ( TITLE-ABS-KEY ( "Temporary Dental Prosthesis" ) OR TITLE-ABS-KEY ( "Dental Fillings, Temporary" ) OR TITLE-ABS-KEY ( "Temporary Dental Fillings" ) OR TITLE-ABS-KEY ( "Temporary Dental Filling" ) OR TITLE-ABS-KEY ( "Dental Filling, Temporary" ) OR TITLE-ABS-KEY ( "Filling, Temporary Dental" ) OR TITLE-ABS-KEY ( "Fillings, Temporary Dental" ) OR TITLE-ABS-KEY ( "Temporary Crown" ) ) ) OR ( ( TITLE-ABS-KEY ( "Temporary Crowns" ) OR TITLE-ABS-KEY ( "Dental veneers" ) OR TITLE-ABS-KEY ( "Veneer, Dental" ) OR TITLE-ABS-KEY ( "Veneers, Dental" ) OR TITLE-ABS-KEY ( "Dental Laminates" ) OR TITLE-ABS-KEY ( "Dental Laminate" ) OR TITLE-ABS-KEY ( "Laminate, Dental" ) OR TITLE-ABS-KEY ( "Laminates, Dental" ) ) ) OR ( ( TITLE-ABS-KEY ( "Dental Veneer" ) OR TITLE-ABS-KEY ( "Ceramic Laminate" ) OR TITLE-ABS-KEY ( "Ceramic Laminates" ) OR TITLE-ABS-KEY ( "Porcelain Laminate" ) OR TITLE-ABS-KEY ( "Porcelain Laminates" ) OR TITLE-ABS-KEY ( "Dental Contact Lenses" ) OR TITLE-ABS-KEY ( "Dental Contact Lens" ) OR TITLE-ABS-KEY ( "Contact Lens" ) ) ) OR ( ( TITLE-ABS-KEY ( "Contact Lenses" ) OR TITLE-ABS-KEY ( "Denture, Partial" ) OR TITLE-ABS-KEY ( "Dentures, Partial" ) OR TITLE-ABS-KEY ( "Partial Denture" ) OR TITLE-ABS-KEY ( "Partial Dentures" ) OR TITLE-ABS-KEY ( "Bridgework, Dental" ) OR TITLE-ABS-KEY ( "Bridgeworks, Dental" ) OR TITLE-ABS-KEY ( "Dental Bridgeworks" ) ) ) OR ( ( TITLE-ABS-KEY ( "Dental Bridgework" ) OR TITLE-ABS-KEY ( "Denture, Partial, Fixed" ) OR TITLE-ABS-KEY ( "Fixed Bridge" ) OR TITLE-ABS-KEY ( "Bridge, Fixed" ) OR TITLE-ABS-KEY ( "Bridges, Fixed" ) OR TITLE-ABS-KEY ( "Fixed Bridges" ) OR TITLE-ABS-KEY ( "Fixed Partial Denture" ) OR TITLE-ABS-KEY ( "Denture, Fixed Partial" ) ) ) OR ( ( TITLE-ABS-KEY ( "Dentures, Fixed Partial" ) OR TITLE-ABS-KEY ( "Fixed Partial Dentures" ) OR TITLE-ABS-KEY ( "Partial Denture, Fixed" ) OR TITLE-ABS-KEY ( "Partial Dentures, Fixed" ) OR TITLE-ABS-KEY ( pontic ) OR TITLE-ABS-KEY ( pontics ) OR TITLE-ABS-KEY ( "Denture, Partial, Fixed, Resin-Bonded" ) OR TITLE-ABS-KEY ( "Resin-Bonded Bridge" ) ) ) OR ( ( TITLE-ABS-KEY ( "Bridge, Resin-Bonded" ) OR TITLE-ABS-KEY ( "Bridges, Resin-Bonded" ) OR TITLE-ABS-KEY ( "Resin Bonded Bridge" ) OR TITLE-ABS-KEY ( "Resin-Bonded Bridges" ) OR TITLE-ABS-KEY ( "Resin-Bonded Acid-Etched Fixed Partial Denture" ) OR TITLE-ABS-KEY ( "Resin Bonded Acid Etched Fixed Partial Denture" ) OR TITLE-ABS-KEY ( "Maryland Bridge" ) OR TITLE-ABS-KEY ( "Bridge, Maryland" ) ) ) OR ( ( TITLE-ABS-KEY ( "Resin-Bonded Fixed Partial Denture" ) OR TITLE-ABS-KEY ( "Resin Bonded Fixed Partial Denture" ) OR TITLE-ABS-KEY ( "Denture, Partial, Immediate" ) OR TITLE-ABS-KEY ( "Denture, Partial, Temporary" ) OR TITLE-ABS-KEY ( "Interim Dental Prosthesis" ) OR TITLE-ABS-KEY ( "Dental Prostheses, Interim" ) OR TITLE-ABS-KEY ( "Interim Dental Prostheses" ) OR TITLE-ABS-KEY ( "Prostheses, Interim Dental" ) ) ) OR ( ( TITLE-ABS-KEY ( "Prosthesis, Interim Dental" ) OR TITLE-ABS-KEY ( "Dental Prosthesis, Interim" ) OR TITLE-ABS-KEY ( "Interim Prosthesis Dental" ) OR TITLE-ABS-KEY ( "Dental, Interim Prosthesis" ) OR TITLE-ABS-KEY ( "Dentals, Interim Prosthesis" ) OR TITLE-ABS-KEY ( "Interim Prosthesis Dentals" ) OR TITLE-ABS-KEY ( "Prosthesis Dental, Interim" ) OR TITLE-ABS-KEY ( "Prosthesis Dentals, Interim" ) ) ) )) AND ((( ( ( TITLE-ABS-KEY ( "Dentin Desensitizing Agents" ) OR TITLE-ABS-KEY ( "Agents, Dentin Desensitizing" ) OR TITLE-ABS-KEY ( "Desensitizing Agents, Dentin" ) OR TITLE-ABS-KEY ( "Dentine Desensitizing Agents" ) OR TITLE-ABS-KEY ( "Agents, Dentine Desensitizing" ) OR TITLE-ABS-KEY ( "Desensitizing Agents, Dentine" ) OR TITLE-ABS-KEY ( "Dentin Desensitizer" ) OR TITLE-ABS-KEY ( "Dentin Desensitizers" ) ) ) OR ( ( TITLE-ABS-KEY ( "Dentine Desensitizer" ) OR TITLE-ABS-KEY ( "Dentine Desensitizers" ) OR TITLE-ABS-KEY ( "Dentin Sensitivity" ) OR TITLE-ABS-KEY ( "Dentin Sensitivities" ) OR TITLE-ABS-KEY ( "Sensitivities, Dentin" ) OR TITLE-ABS-KEY ( "Sensitivity, Dentin" ) OR TITLE-ABS-KEY ( "Dentine Hypersensitivity" ) OR TITLE-ABS-KEY ( "Dentine Hypersensitivities" ) ) ) OR ( ( TITLE-ABS-KEY ( "Hypersensitivities, Dentine" ) OR TITLE-ABS-KEY ( "Hypersensitivity, Dentine" ) OR TITLE-ABS-KEY ( "Dentine Sensitivity" ) OR TITLE-ABS-KEY ( "Dentine Sensitivities" ) OR TITLE-ABS-KEY ( "Sensitivities, Dentine" ) OR TITLE-ABS-KEY ( "Sensitivity, Dentine" ) OR TITLE-ABS-KEY ( "Tooth Sensitivity" ) OR TITLE-ABS-KEY ( "Sensitivities, Tooth" ) ) ) OR ( ( TITLE-ABS-KEY ( "Sensitivity, Tooth" ) OR TITLE-ABS-KEY ( "Tooth Sensitivities" ) OR TITLE-ABS-KEY ( "Dentin Hypersensitivity" ) OR TITLE-ABS-KEY ( "Dentin Hypersensitivities" ) OR TITLE-ABS-KEY ( "Hypersensitivities, Dentin" ) OR TITLE-ABS-KEY ( "Hypersensitivity, Dentin" ) OR TITLE-ABS-KEY ( "Dentin Permeability" ) OR TITLE-ABS-KEY ( "Dentin Permeabilities" ) ) ) OR ( ( TITLE-ABS-KEY ( "Permeabilities, Dentin" ) OR TITLE-ABS-KEY ( "Permeability, Dentin" ) OR TITLE-ABS-KEY ( "Dentine Permeability" ) OR TITLE-ABS-KEY ( "Dentine Permeabilities" ) OR TITLE-ABS-KEY ( "Permeabilities, Dentine" ) OR TITLE-ABS-KEY ( "Permeability, Dentine" ) OR TITLE-ABS-KEY ( "Tooth Permeability" ) OR TITLE-ABS-KEY ( "Permeability, Tooth" ) ) ) OR ( ( TITLE-ABS-KEY ( "Permeabilities, Tooth" ) OR TITLE-ABS-KEY ( "Tooth Permeabilities" ) OR TITLE-ABS-KEY ( "Dental Enamel Permeability" ) OR TITLE-ABS-KEY ( "Permeability, Dental Enamel" ) OR TITLE-ABS-KEY ( "Enamel Permeability, Dental" ) OR TITLE-ABS-KEY ( "Dentin immediate sealing" ) OR TITLE-ABS-KEY ( "Immediate dentin sealing" ) OR TITLE-ABS-KEY ( "Dentine immediate sealing" ) ) ) OR ( ( TITLE-ABS-KEY ( "Immediate dentine sealing" ) OR TITLE-ABS-KEY ( "Dentin sealing" ) OR TITLE-ABS-KEY ( "Dentine sealing" ) OR TITLE-ABS-KEY ( "Dentin seal" ) OR TITLE-ABS-KEY ( "Dentine seal" ) OR TITLE-ABS-KEY ( ids ) OR TITLE-ABS-KEY ( pre-hybridization ) OR TITLE-ABS-KEY ( "Pre hybridization" ) ) ) OR ( ( TITLE-ABS-KEY ( prehybridization ) OR TITLE-ABS-KEY ( pre-hybridisation ) OR TITLE-ABS-KEY ( "Pre hybridization" ) OR TITLE-ABS-KEY ( prehybridisation ) ) ) )) OR (TITLE-ABS-KEY("Resin coating" OR resin-coating))) AND (( ( ( TITLE-ABS-KEY ( clinical ) OR TITLE-ABS-KEY ( randomized ) OR TITLE-ABS-KEY ( "Intervention Study" ) OR TITLE-ABS-KEY ( "Intervention Studies" ) OR TITLE-ABS-KEY ( controlled AND trial* ) OR TITLE-ABS-KEY ( prospective ) OR TITLE-ABS-KEY ( follow-up* ) OR TITLE-ABS-KEY ( "Follow Up" ) ) ) OR ( ( TITLE-ABS-KEY ( trial* ) OR TITLE-ABS-KEY ( longitudinal ) OR TITLE-ABS-KEY ( quasi-experimental ) OR TITLE-ABS-KEY ( non-randomized ) OR TITLE-ABS-KEY ( nonrandomized ) ) ) )) not INDEX ( medline ) |
| EMBASE (76) | #1  crowns:ti,ab,kw OR crown:ti,ab,kw OR 'dental crowns':ti,ab,kw OR 'dental crown':ti,ab,kw OR 'crown, dental':ti,ab,kw OR 'crowns, dental':ti,ab,kw OR 'prosthetic crown':ti,ab,kw OR 'single crown':ti,ab,kw OR 'dental prosthesis retention':ti,ab,kw OR 'prosthesis retention, dental':ti,ab,kw OR 'retention, dental prosthesis':ti,ab,kw OR 'dental prosthesis':ti,ab,kw OR 'prosthesis, dental':ti,ab,kw OR 'dental prostheses':ti,ab,kw OR 'prostheses, dental':ti,ab,kw OR 'dental prosthodontics':ti,ab,kw OR 'tooth prosthesis':ti,ab,kw OR 'tooth prostheses':ti,ab,kw OR 'tooth prosthodontics':ti,ab,kw OR 'teeth prosthesis':ti,ab,kw OR 'teeth prostheses':ti,ab,kw OR 'teeth prosthodontics':ti,ab,kw OR 'dental restoration failure':ti,ab,kw OR 'failure, dental restoration':ti,ab,kw OR 'restoration failure, dental':ti,ab,kw OR 'restoration failures, dental':ti,ab,kw OR 'dental restoration failures':ti,ab,kw OR 'failures, dental restoration':ti,ab,kw OR 'dental prosthesis failure':ti,ab,kw OR 'failure, dental prosthesis':ti,ab,kw OR 'prosthesis failure, dental':ti,ab,kw OR 'prosthesis failures, dental':ti,ab,kw OR 'dental prosthesis failures':ti,ab,kw OR 'failures, dental prosthesis':ti,ab,kw OR 'dental restoration, permanent':ti,ab,kw OR 'restorations, permanent dental':ti,ab,kw OR 'permanent dental restorations':ti,ab,kw OR 'restoration, permanent dental':ti,ab,kw OR 'dental restorations, permanent':ti,ab,kw OR 'permanent dental restoration':ti,ab,kw OR 'dental permanent fillings':ti,ab,kw OR 'fillings, permanent dental':ti,ab,kw OR 'permanent dental fillings':ti,ab,kw OR 'permanent fillings, dental':ti,ab,kw OR 'permanent filling, dental':ti,ab,kw OR 'dental filling, permanent':ti,ab,kw OR 'dental permanent filling':ti,ab,kw OR 'filling, dental permanent':ti,ab,kw OR 'filling, permanent dental':ti,ab,kw OR 'permanent dental filling':ti,ab,kw OR 'fillings, dental permanent':ti,ab,kw OR 'dental fillings, permanent':ti,ab,kw OR inlays:ti,ab,kw OR inlay:ti,ab,kw OR 'inlay, dental':ti,ab,kw OR 'inlays, dental':ti,ab,kw OR 'dental inlays':ti,ab,kw OR 'dental onlays':ti,ab,kw OR 'dental inlay':ti,ab,kw OR 'dental onlay':ti,ab,kw OR 'onlay, dental':ti,ab,kw OR 'onlays, dental':ti,ab,kw OR onlays:ti,ab,kw OR onlay:ti,ab,kw OR 'tooth inlay':ti,ab,kw OR 'tooth inlays':ti,ab,kw OR 'tooth onlay':ti,ab,kw OR 'tooth onlays':ti,ab,kw OR overlay:ti,ab,kw OR overlays:ti,ab,kw OR 'dental overlay':ti,ab,kw OR 'dental overlays':ti,ab,kw OR 'tooth overlay':ti,ab,kw OR 'tooth overlays':ti,ab,kw OR 'dental restoration, temporary':ti,ab,kw OR 'temporary dental restorations':ti,ab,kw OR 'restorations, temporary dental':ti,ab,kw OR 'temporary dental restoration':ti,ab,kw OR 'dental restorations, temporary':ti,ab,kw OR 'restoration, temporary dental':ti,ab,kw OR 'dental prosthesis, temporary':ti,ab,kw OR 'dental prostheses, temporary':ti,ab,kw OR 'prosthesis, temporary dental':ti,ab,kw OR 'temporary dental prostheses':ti,ab,kw OR 'temporary dental prosthesis':ti,ab,kw OR 'dental fillings, temporary':ti,ab,kw OR 'temporary dental fillings':ti,ab,kw OR 'temporary dental filling':ti,ab,kw OR 'dental filling, temporary':ti,ab,kw OR 'filling, temporary dental':ti,ab,kw OR 'fillings, temporary dental':ti,ab,kw OR 'temporary crown':ti,ab,kw OR 'temporary crowns':ti,ab,kw OR 'dental veneers':ti,ab,kw OR 'veneer, dental':ti,ab,kw OR 'veneers, dental':ti,ab,kw OR 'dental laminates':ti,ab,kw OR 'dental laminate':ti,ab,kw OR 'laminate, dental':ti,ab,kw OR 'laminates, dental':ti,ab,kw OR 'dental veneer':ti,ab,kw OR 'ceramic laminate':ti,ab,kw OR 'ceramic laminates':ti,ab,kw OR 'porcelain laminate':ti,ab,kw OR 'porcelain laminates':ti,ab,kw OR 'dental contact lenses':ti,ab,kw OR 'dental contact lens':ti,ab,kw OR 'contact lens':ti,ab,kw OR 'contact lenses':ti,ab,kw OR 'denture, partial':ti,ab,kw OR 'dentures, partial':ti,ab,kw OR 'partial denture':ti,ab,kw OR 'partial dentures':ti,ab,kw OR 'bridgework, dental':ti,ab,kw OR 'bridgeworks, dental':ti,ab,kw OR 'dental bridgeworks':ti,ab,kw OR 'dental bridgework':ti,ab,kw OR 'denture, partial, fixed':ti,ab,kw OR 'fixed bridge':ti,ab,kw OR 'bridge, fixed':ti,ab,kw OR 'bridges, fixed':ti,ab,kw OR 'fixed bridges':ti,ab,kw OR 'fixed partial denture':ti,ab,kw OR 'denture, fixed partial':ti,ab,kw OR 'dentures, fixed partial':ti,ab,kw OR 'fixed partial dentures':ti,ab,kw OR 'partial denture, fixed':ti,ab,kw OR 'partial dentures, fixed':ti,ab,kw OR pontic:ti,ab,kw OR pontics:ti,ab,kw OR 'denture, partial, fixed, resin-bonded':ti,ab,kw OR 'resin-bonded bridge':ti,ab,kw OR 'bridge, resin-bonded':ti,ab,kw OR 'bridges, resin-bonded':ti,ab,kw OR 'resin bonded bridge':ti,ab,kw OR 'resin-bonded bridges':ti,ab,kw OR 'resin-bonded acid-etched fixed partial denture':ti,ab,kw OR 'resin bonded acid etched fixed partial denture':ti,ab,kw OR 'maryland bridge':ti,ab,kw OR 'bridge, maryland':ti,ab,kw OR 'resin-bonded fixed partial denture':ti,ab,kw OR 'resin bonded fixed partial denture':ti,ab,kw OR 'denture, partial, immediate':ti,ab,kw OR 'denture, partial, temporary':ti,ab,kw OR 'interim dental prosthesis':ti,ab,kw OR 'dental prostheses, interim':ti,ab,kw OR 'interim dental prostheses':ti,ab,kw OR 'prostheses, interim dental':ti,ab,kw OR 'prosthesis, interim dental':ti,ab,kw OR 'dental prosthesis, interim':ti,ab,kw OR 'interim prosthesis dental':ti,ab,kw OR 'dental, interim prosthesis':ti,ab,kw OR 'dentals, interim prosthesis':ti,ab,kw OR 'interim prosthesis dentals':ti,ab,kw OR 'prosthesis dental, interim':ti,ab,kw OR 'prosthesis dentals, interim':ti,ab,kw  #2  'dentin desensitizing agents':ti,ab,kw OR 'agents, dentin desensitizing':ti,ab,kw OR 'desensitizing agents, dentin':ti,ab,kw OR 'dentine desensitizing agents':ti,ab,kw OR 'agents, dentine desensitizing':ti,ab,kw OR 'desensitizing agents, dentine':ti,ab,kw OR 'dentin desensitizer':ti,ab,kw OR 'dentin desensitizers':ti,ab,kw OR 'dentine desensitizer':ti,ab,kw OR 'dentine desensitizers':ti,ab,kw OR 'dentin sensitivity':ti,ab,kw OR 'dentin sensitivities':ti,ab,kw OR 'sensitivities, dentin':ti,ab,kw OR 'sensitivity, dentin':ti,ab,kw OR 'dentine hypersensitivity':ti,ab,kw OR 'dentine hypersensitivities':ti,ab,kw OR 'hypersensitivities, dentine':ti,ab,kw OR 'hypersensitivity, dentine':ti,ab,kw OR 'dentine sensitivity':ti,ab,kw OR 'dentine sensitivities':ti,ab,kw OR 'sensitivities, dentine':ti,ab,kw OR 'sensitivity, dentine':ti,ab,kw OR 'tooth sensitivity':ti,ab,kw OR 'sensitivities, tooth':ti,ab,kw OR 'sensitivity, tooth':ti,ab,kw OR 'tooth sensitivities':ti,ab,kw OR 'dentin hypersensitivity':ti,ab,kw OR 'dentin hypersensitivities':ti,ab,kw OR 'hypersensitivities, dentin':ti,ab,kw OR 'hypersensitivity, dentin':ti,ab,kw OR 'dentin permeability':ti,ab,kw OR 'dentin permeabilities':ti,ab,kw OR 'permeabilities, dentin':ti,ab,kw OR 'permeability, dentin':ti,ab,kw OR 'dentine permeability':ti,ab,kw OR 'dentine permeabilities':ti,ab,kw OR 'permeabilities, dentine':ti,ab,kw OR 'permeability, dentine':ti,ab,kw OR 'tooth permeability':ti,ab,kw OR 'permeability, tooth':ti,ab,kw OR 'permeabilities, tooth':ti,ab,kw OR 'tooth permeabilities':ti,ab,kw OR 'dental enamel permeability':ti,ab,kw OR 'permeability, dental enamel':ti,ab,kw OR 'enamel permeability, dental':ti,ab,kw OR 'dentin immediate sealing':ti,ab,kw OR 'immediate dentin sealing':ti,ab,kw OR 'dentine immediate sealing':ti,ab,kw OR 'immediate dentine sealing':ti,ab,kw OR 'dentin sealing':ti,ab,kw OR 'dentine sealing':ti,ab,kw OR 'dentin seal':ti,ab,kw OR 'dentine seal':ti,ab,kw OR ids:ti,ab,kw OR prehybridization:ti,ab,kw OR 'pre hybridisation':ti,ab,kw OR 'pre hybridization':ti,ab,kw OR prehybridisation:ti,ab,kw OR 'resin coating':ti,ab,kw OR resin-coating:ti,ab,kw  #3  clinical:ti,ab,kw OR randomized:ti,ab,kw OR 'intervention study':ti,ab,kw OR 'intervention studies':ti,ab,kw OR 'controlled trial*':ti,ab,kw OR prospective:ti,ab,kw OR 'follow up*':ti,ab,kw OR 'follow up':ti,ab,kw OR trial*:ti,ab,kw OR longitudinal:ti,ab,kw OR 'quasi experimental':ti,ab,kw OR 'non randomized':ti,ab,kw OR nonrandomized:ti,ab,kw  #1 AND #2 AND #3 |
| ReBEC (0) | Search: immediate dentin sealing AND dental prosthesis (0)  Search: resin coating AND dental prosthesis (0) |
| Open Grey (0) | Search: immediate dentin sealing AND dental prosthesis (0)  Search: resin coating AND dental prosthesis (0) |
| Clinical Trials (51) | Search: dentin sealing OR dentin sensitivity OR immediate dentin sealing OR IDS OR resin coating OR resin-coating \| decayed tooth OR fractured tooth OR crown OR inlay OR onlay OR veneer OR laminate OR prosthesis OR fixed partial denture |
