# Supplementary material for: Does immediate dentin sealing influence postoperative sensitivity in teeth restored with indirect restorations? A systematic review and meta‐analysis
Source: J Esthet Restor Dent. 2021 Dec 3;34(1):55–64. doi: 10.1111/jerd.12841 (PMC9300029; doi:10.1111/jerd.12841)
Supplement: Supplementary file 2 — Table S2 Information about the studies included in this systematic review and meta‐analysis [file JERD-34-55-s001.docx]

Table 2. Information about the studies included in this systematic review and meta-analysis.

| Author, year, and location | Study Design | Participants  (age range in years) | Indirect restorations (tooth and type) | Dentin sealing approach | | Evaluation criteria (outcomes) | Follow-up in months (recall %) | Success rate n (each treatment) | Failure rate n (each treatment) | Statistical analysis |
| --- | --- | --- | --- | --- | --- | --- | --- | --- | --- | --- |
|  |  |  |  | IDS  (n and adhesive) | DDS  (n) |  |  |  |  |  |
| Gresnigt et al., 2019 (The Netherlands) | Clinical trial | 118 (18-78 / mean 42.1) | 444 indirect ceramic laminate veeners (156 in central incisors, 125 in lateral incisors, and 103 in canines) | Not clearly stated (Optibond FL Prime + Optibond FL adhesive) | Not clearly stated | Modified USPHS (adaptation, color match, marginal discoloration, surface roughness, fracture of restoration, fracture of tooth, wear of restoration, wear of antagonist, caries, postoperative sensitivity)  Light transillumination (internal microcracks) | Baseline, 3 months, 12 months, and 11 years (100% for baseline, 88% for 11 years) | IDS  At 11 years  Overall survival rate: 96.4%  DDS  At 11 years  Overall survival rate: 81.8% | IDS  At 11 years  Overall failure rate: 3.6%  DDS  At 11 years  Overall survival rate: 18.2% | Kaplan–Meier and Log Rank (Mantel–Cox) - alfa=0.05  Significant difference between IDS and DDS survival rates (p=0.017) |
| Hu & Qingdang, 2010 (China) | Clinical trial | 25 (20-30 years) | 50 abutment crowns (25 premolars and 25 molars) | 25 (Prime & Bond, Dentsply) | 25 | Sensitivity discomfort interval scale (0=no pain, 1=mild pain, 2=moderate pain, 3=severe pain, 4=intolerable pain) | 1 week, 1 month, 6 months, 12 months, and 24 months | SCORE 0 (NO PAIN)  IDS  Sensitivity  1 week: 17/25  1 month: 18/25  6 months: 21/25  12 months: 23/25  24 months: 23/25  DDS  Sensitivity  1 week: 7/25  1 month: 8/25  6 months: 17/25  12 months: 22/25  24 months: 22/25 | SCORES 1, 2, 3, AND 4 (MILD, MODERATE, SEVERE, AND INTOLERABLE PAIN)  IDS  Sensitivity  1 week: 8/25  1 month: 7/25  6 months: 4/25  12 months: 2/25  24 months: 2/25  DDS  Sensitivity  1 week: 18/25  1 month: 17/25  6 months: 8/25  12 months: 3/25  24 months: 3/25 | Sign test (alfa=0.05)  The sensitivity scores for teeth in Group A were statistically significantly lower than those in the Group B 1 week and 1 month after cementation (P < .05), whereas there was no significant difference between Groups A and B at 6, 12, and 24 months after cementation (P > .05). |
| van den Breemer et al., 2019a (The Netherlands) | Randomized controlled, single-blind clinical trial with within-subject comparison. | 30 (mean 54) | 60 indirect partial ceramic restoration (molar) | 30 (Clearfil  SE Primer and Adhesive + Clearfil Majesty Flow) | 30 | Objective tooth sensitivity measurement by clinical exam  Subjective tooth sensitivity (patient’s indication of sensitivity)  VAS scale for experienced discomfort (from 0=none to 10=very painful)  VAS scale (color, shape, ability to chew) for patient satisfaction from 0=very satisfied to 10=not satisfied | 1 week, 3 months, 12 months | IDS  Retention  100% (all restorations in situ)  Vitality  100%  DDS  Retention  100% (all restorations in situ)  Vitality  100% | IDS  Retention  0% (no restorations debonded)  Vitality  0%  DDS  Retention  0% (no restorations debonded)  Vitality  0% | McNemar test  Chi-square test  Wilcoxon signed rank tests  p values less than 0.01 were considered to be statistically significant in all tests  There was also no significant difference between IDS and DDS (p.0.01) for all items in the questionnaire.  Patient satisfaction according to the VAS scores  was not significantly different between the IDS and DDS groups for all items in the questionnaire at all time points (p.0.01) |
| van den Breemer et al., 2019b (The Netherlands) | Randomized controlled, single blind clinical trial with within-subject comparison. | 30 (mean 54) | 60 indirect partial ceramic restoration (molar) | 30 (Clearfil  SE Primer and Adhesive + Clearfil Majesty Flow) | 30 | FDI criteria (surface luster, staining surface / margin, color match and translucency, esthetic anatomical form, fracture of material and retention, marginal adaptation, occlusal contour wear qualitatively / quantitatively, approximal anatomic form contact point contour, radiographic examination, patient’s view, postoperative (hyper-)sensitivity and tooth vitality, recurrence of caries, erosion, abfraction, tooth integrity (enamel cracks, tooth fractures), periodontal response (compared to a reference tooth), adjacent mucosa, and oral and general health) | 1 week, 12 months, 36 months (100% recall rate) | Overall survival rates at 36 months  IDS: 100%  DDS: 96.7%  SCORES 1, 2, 3 (SUCCESS)  IDS  Surface luster  1 week: 30/30  12 months: 30/30  36 months: 30/30  Staining surface / margin  1 week: 30/30  12 months: 30/30  36 months: 30/30  Color match and translucency  1 week: 30/30  12 months: 30/30  36 months: 30/30  Esthetic anatomical form  1 week: 30/30  12 months: 30/30  36 months: 30/30  Fracture of material and retention  1 week: 30/30  12 months: 30/30  36 months: 30/30  Marginal Adaptation  1 week: 30/30  12 months: 30/30  36 months: 30/30  Occlusal contour wear qualitatively / quantitatively  1 week: 30/30  12 months: 30/30  36 months: 29/30  Approximal anatomical form contact point / contour  1 week: 30/30  12 months: 30/30  36 months: 30/30  Radiographic examination  1 week: 30/30  12 months: 30/30  36 months: 30/30  Patient’s view  1 week: 30/30  12 months: 30/30  36 months: 30/30  Postoperative (hyper-)sensitivity and tooth vitality  1 week: 30/30  12 months: 30/30  36 months: 30/30  Recurrence of caries, erosion, abfraction  1 week: 30/30  12 months: 30/30  36 months: 29/30  Tooth integrity (enamel cracks, tooth fractures)  1 week: 30/30  12 months: 30/30  36 months: 30/30  Periodontal response (compared to a reference tooth)  1 week: 30/30  12 months: 29/30  36 months: 30/30  Adjacent mucosa  1 week: 30/30  12 months: 30/30  36 months: 30/30  Oral and general health  1 week: 30/30  12 months: 30/30  36 months: 30/30  Overall success rate (restorations with scores 1, 2, 3)  36 months: 86.7%  DDS  Surface luster  1 week: 30/30  12 months: 30/30  36 months: 30/30  Staining surface / margin  1 week: 30/30  12 months: 30/30  36 months: 30/30  Color match and translucency  1 week: 30/30  12 months: 30/30  36 months: 30/30  Esthetic anatomical form  1 week: 30/30  12 months: 30/30  36 months: 30/30  Fracture of material and retention  1 week: 30/30  12 months: 30/30  36 months: 27/30  Marginal Adaptation  1 week: 30/30  12 months: 30/30  36 months: 30/30  Occlusal contour wear qualitatively / quantitatively  1 week: 30/30  12 months: 30/30  36 months: 30/30  Approximal anatomical form contact point / contour  1 week: 30/30  12 months: 30/30  36 months: 30/30  Radiographic examination  1 week: 30/30  12 months: 30/30  36 months: 30/30  Patient’s view  1 week: 30/30  12 months: 30/30  36 months: 29/30  Postoperative (hyper-)sensitivity and tooth vitality  1 week: 30/30  12 months: 30/30  36 months: 30/30  Recurrence of caries, erosion, abfraction  1 week: 30/30  12 months: 30/30  36 months: 29/30  Tooth integrity (enamel cracks, tooth fractures)  1 week: 30/30  12 months: 29/30  36 months: 28/30  Periodontal response (compared to a reference tooth)  1 week: 30/30  12 months: 29/30  36 months: 30/30  Adjacent mucosa  1 week: 30/30  12 months: 30/30  36 months: 30/30  Oral and general health  1 week: 30/30  12 months: 30/30  36 months: 30/30  Overall success rate (restorations with scores 1, 2, 3)  36 months: 83.3% | Overall failure rates at 36 months  IDS: 0%  DDS: 3.3%  SCORES 4 (REPAIRABLE FAILURE), AND 5 (COMPLETE FAILURE)  IDS  Surface luster  1 week: 0/30  12 months: 0/30  36 months: 0/30  Staining surface / margin  1 week: 0/30  12 months: 0/30  36 months: 0/30  Color match and translucency  1 week: 0/30  12 months: 0/30  36 months: 0/30  Esthetic anatomical form  1 week: 0/30  12 months: 0/30  36 months: 0/30  Fracture of material and retention  1 week: 0/30  12 months: 0/30  36 months: 0/30  Marginal Adaptation  1 week: 0/30  12 months: 0/30  36 months: 0/30  Occlusal contour wear qualitatively / quantitatively  1 week: 0/30  12 months: 0/30  36 months: 1/30  Approximal anatomical form contact point / contour  1 week: 0/30  12 months: 0/30  36 months: 0/30  Radiographic examination  1 week: 0/30  12 months: 0/30  36 months: 0/30  Patient’s view  1 week: 0/30  12 months: 0/30  36 months: 0/30  Postoperative (hyper-)sensitivity and tooth vitality  1 week: 0/30  12 months: 0/30  36 months: 0/30  Recurrence of caries, erosion, abfraction  1 week: 0/30  12 months: 0/30  36 months: 1/30  Tooth integrity (enamel cracks, tooth fractures)  1 week: 0/30  12 months: 0/30  36 months: 0/30  Periodontal response (compared to a reference tooth)  1 week: 0/30  12 months: 1/30  36 months: 0/30  Adjacent mucosa  1 week: 0/30  12 months: 0/30  36 months: 0/30  Oral and general health  1 week: 0/30  12 months: 0/30  36 months: 0/30  Overall failure rate (restorations with scores 4, 5)  36 months: 13.3%  DDS  Surface luster  1 week: 0/30  12 months: 0/30  36 months: 0/30  Staining surface / margin  1 week: 0/30  12 months: 0/30  36 months: 0/30  Color match and translucency  1 week: 0/30  12 months: 0/30  36 months: 0/30  Esthetic anatomical form  1 week: 0/30  12 months: 0/30  36 months: 0/30  Fracture of material and retention  1 week: 0/30  12 months: 0/30  36 months: 3/30  Marginal Adaptation  1 week: 0/30  12 months: 0/30  36 months: 0/30  Occlusal contour wear qualitatively / quantitatively  1 week: 0/30  12 months: 0/30  36 months: 0/30  Approximal anatomical form contact point / contour  1 week: 0/30  12 months: 0/30  36 months: 0/30  Radiographic examination  1 week: 0/30  12 months: 0/30  36 months: 0/30  Patient’s view  1 week: 0/30  12 months: 0/30  36 months: 1/30  Postoperative (hyper-)sensitivity and tooth vitality  1 week: 0/30  12 months: 0/30  36 months: 0/30  Recurrence of caries, erosion, abfraction  1 week: 0/30  12 months: 0/30  36 months: 1/30  Tooth integrity (enamel cracks, tooth fractures)  1 week: 0/30  12 months: 1/30  36 months: 2/30  Periodontal response (compared to a reference tooth)  1 week: 0/30  12 months: 1/30  36 months: 0/30  Adjacent mucosa  1 week: 0/30  12 months: 0/30  36 months: 0/30  Oral and general health  1 week: 0/30  12 months: 0/30  36 months: 0/30  Overall failure rate (restorations with scores 4, 5)  36 months: 16.7% | Data were analyzed using Kaplan-Meier (Log Rank (Mantel-Cox)) tests to obtain the overall success and survival rates in relation to observation time, and Wilcoxon test and McNemar test were used to test differences in the overall quality of survival. The alpha level was set at 0.05 in all tests.  The survival rates for IDS and DDS were 100% and 96.7% respectively (not significantly different, p=0.32; KaplanMeier, Log Rank (Mantel-Cox), CI=95%). The overall success rate (FDI criteria score 1–3, restorations without  any adverse effects, n=51) after 36 months is 85% (Fig. 6), not different for the IDS and DDS group (86.7% versus 83.3%, p=0.7; Kaplan-Meier, Log Rank (Mantel-Cox), CI=95%). No statistically significant differences were found between IDS and DDS restorations on any follow-up timepoints for any of the FDI criteria (Wilcoxon, McNemar, p > 0.05). |
